# Modeling inter-trial variability of pointing movements during visuomotor adaptation

**DOI:** 10.1007/s00422-021-00858-w

**Published:** 2021-02-11

**Authors:** Thomas Eggert, Denise Y. P. Henriques, Bernard M. ’t Hart, Andreas Straube

**Affiliations:** 1 Department of Neurology, University Hospital, LMU Munich, Fraunhoferstr. 20, 82152 Planegg, Martinsried, Germany; 2 School of Kinesiology and Health Science, Centre for Vision Research, York University, 4700 Keele Street, Toronto, ON M3J 1P3 Canada; 3 Centre for Vision Research, York University, 4700 Keele Street, Toronto, ON M3J 1P3 Canada; 4 Department of Neurology and German Center for Vertigo and Balance Disorders-DSGZ, University Hospital LMU, Munich, Marchioninistr. 15, 81377 Munich, Germany

**Keywords:** Human, Motor control, Reaching movements, Adaptation, System identification, Kalman filter

## Abstract

Trial-to-trial variability during visuomotor adaptation is usually explained as the result of two different sources, planning noise and execution noise. The estimation of the underlying variance parameters from observations involving varying feedback conditions cannot be achieved by standard techniques (Kalman filter) because they do not account for recursive noise propagation in a closed-loop system. We therefore developed a method to compute the exact likelihood of the output of a time-discrete and linear adaptation system as has been used to model visuomotor adaptation (Smith et al. in PLoS Biol 4(6):e179, 2006), observed under closed-loop and error-clamp conditions. We identified the variance parameters by maximizing this likelihood and compared the model prediction of the time course of variance and autocovariance with empiric data. The observed increase in variability during the early training phase could not be explained by planning noise and execution noise with constant variances. Extending the model by signal-dependent components of either execution noise or planning noise showed that the observed temporal changes of the trial-to-trial variability can be modeled by signal-dependent planning noise rather than signal-dependent execution noise. Comparing the variance time course between different training schedules showed that the signal-dependent increase of planning variance was specific for the fast adapting mechanism, whereas the assumption of constant planning variance was sufficient for the slow adapting mechanisms.

## Introduction

Programming visually guided movements requires associating visual errors with the appropriate motor corrections. For example, the visual representations of the distance and direction of a target from the hand can guide a pointing movement to the target. Random and unexpected errors of such movements can be corrected by visual feedback used either for driving a series of visually guided corrective movements or by online corrections integrated in the ongoing primary movement. In contrast, repeated occurrence of systematic errors gradually changes the relation between the visual stimulus and the feedforward component of the motor command. This gradual distortion of the mapping of the visual space onto the motor space is called motor adaptation and represents a basic form of motor learning. Motor adaptation was investigated in different motor modalities and error types, such as prism adaptation (Held and Schlank 1959), saccade adaptation (McLaughlin 1967), force field adaptation (Lackner and Dizio 1994; Shadmehr and Mussa-Ivaldi 1994), and adaptation to visuomotor rotation (Cunningham 1989).

### Present state of modeling adaptation dynamics

**Fig. 1:**
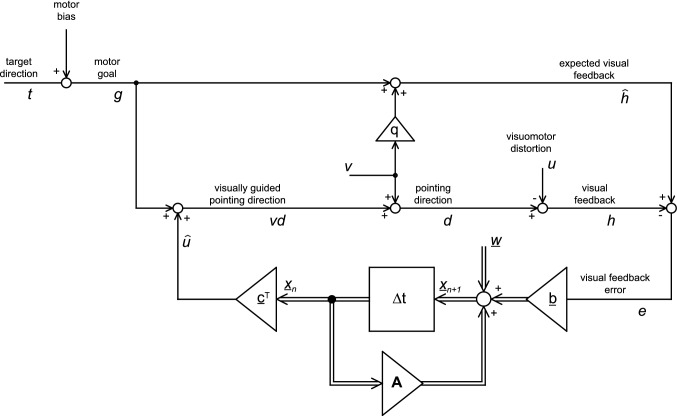
Structure of a classical multi-rate adaptive system, modeled as a time-discrete linear filter. The visuomotor distortion (*u*) is the input, and the pointing direction (*d*) is the output of this system. The two components of the vector *x* represent the slow and fast adapted memory states. These are equally weighted by the scalar product with the vector *c* = [1; 1] and added to the motor goal (*g*) to form the adapted visually guided pointing direction (*vd*). This internal motor plan is superimposed with other components (*v*) contributing to the executed pointing direction (d). These non-visual components include planned exploration, peripheral motor noise, and potential external mechanical perturbations. In the current experiment, they are not determined by the experimental stimulus of the experiment and are therefore subsumed under the term “execution noise” (*v*). The visual error (*e*) is the input to the adaptation dynamics modeled as a 2D state-space model with error sensitivities $\underline{b}=\left[{b}_{s};{b}_{f}\right]$ and error sensitivities $${\varvec{A}}=\left[\begin{array}{cc}{a}_{s}& 0\\ 0& {a}_{f}\end{array}\right]$$. The model is identical with the one proposed by (Smith et al. 2006) except the additional optional feature (for *q* > 0) that the execution noise *v* can partly be accounted for by the expected visual feedback

Previous studies modeled force-field adaptation (Smith et al. 2006), saccade adaptation (Chen-Harris et al. 2008; Ethier et al. 2008), and visuomotor adaptation (McDougle et al. 2015) by the linear dynamics of one or two adaptive memory states driven by the experienced errors. The two essential features of linear adaptation dynamics are the following. First, small errors induce small adaptive changes and larger errors induce larger adaptive changes. The ratio between the adaptive change and the error is called *error sensitivity*. Second, acquired adaptive changes decrease in the absence of errors, faster for large than for small errors. The fraction of the adaptation that is retained after a time step is called the *retention rate*. In the simplest case, with only a single memory state, these dynamics predict exponential time courses for learning and forgetting. The studies mentioned above showed that motor adaptation involves two components with different dynamics, a fast component with a large error sensitivity and a small retention rate, and a slow component with a small error sensitivity and a large retention rate (Fig. 1). This two-rate model (described in detail in “Appendix A1”) is very successful in explaining an effect that occurs during error-clamp trials (i.e., when errors are clamped to zero by the experimenter) after a reversal training period. Under this condition, a spontaneous recovery of the adaptive changes toward the initial adaptation direction is observed. This so-called *rebound effect* was observed in force-field adaptation (Smith et al. 2006), in saccade adaptation (Ethier et al. 2008), and in adaptations to visuomotor rotations (McDougle et al. 2015) and is adequately predicted by the two-rate model.

This classical model of sensorimotor adaptation has two different sources of trial-to-trial variability: The first source is called execution noise, subsuming all motor effects on the initial pointing direction that are unrelated to the visually guided motor plan. The second source of trial-to-trial variability is additive noise on the internal memory states reflecting the variability of the fast and slowly adapting components of the visually guided motor plan. Therefore, we call this second noise component *planning noise*. The distinction was made originally by van Beers (2009) who investigated the trial-to-trial variability of repeated visually guided pointing movements. In his study, the autocorrelation (lag 1) of repeated pointing movements was close to zero and differed significantly from the negative autocorrelation expected if, at positive error sensitivities ($\underline{b}$ in Fig. 1), the trial-to-trial noise were exclusively due to peripheral execution noise ($v$ in Fig. 1). In contrast, planning noise entering at the level of the internal memory states (noise vector $\underline{w}$ in Fig. 1) induces a positive autocorrelation. Qualitatively, these predictions follow directly from the fact that execution noise transfers with a negative sign to the next trial (because of the adaptive error correction), whereas the planning noise transfers with a positive sign to the next trial (since the retention factors ${\varvec{A}}$ are positive). Therefore, van Beers (2009) could explain the missing negative autocorrelation by the superposition of execution noise and planning noise. These experiments did not involve adaptation to artificially altered visual feedback (*u* in Fig. 1), but the distinction between execution noise and planning noise was also made in modeling trial-to-trial variability in visuomotor adaptation (Zarahn et al. 2008; Albert and Shadmehr 2017). Under the assumption of linear adaptation dynamics, this seems a natural generalization since in linear systems the noise superimposes additively with the deterministic inputs and is therefore not expected to depend on additional input such as an externally applied visuomotor distortion.

The model shown in Fig. 1 is most generally described by the following formulation:

1a $$ \underline {x}_{n + 1} = {\varvec{A}}_{n} \user2{ }\underline {x}_{n} + \underline {b}_{n} { }\left( {z_{n} + {\upgamma }_{n} v_{n} } \right) + {\varvec{D}}_{n} \user2{ }\underline {w}_{n} $$

1b $$ y_{n} = \underline {c}^{T} \underline {x}_{n} + v_{n} . $$

The detailed definitions of this system and of the involved variables are provided in “Appendix A1” (Eq. A16a–f). **Here we only briefly mention the most important ones: The system output ${y}_{n}$ denotes the relative pointing direction with respect to baseline and ${z}_{n}$ denotes the deterministic input dependent on either the visuomotor distortion (${u}_{n}$) in closed-loop trials or on the direction of the cursor movement (${h}_{n}$) in error-clamp trials. A linear combination of two noise signals (${v}_{n}$, ${w}_{n}$) contaminates the update of the system states ${\underline{x}}_{n}$ (Eq. 1a), whereas the output equation (Eq. 1b) is only affected by ${v}_{n}$. The two noise signals were assumed to be independent:

2 $$ \left[ {\begin{array}{*{20}c} {v_{n} } \\ {\underline {w}_{n} } \\ \end{array} } \right]\sim N\left( {\left[ {\begin{array}{*{20}c} 0 \\ {\underline {0} } \\ \end{array} } \right],\left[ {\begin{array}{*{20}c} {\sigma^{2} } & {\underline {0}^{T} } \\ {\underline {0} } & {\varvec{W}} \\ \end{array} } \right]} \right). $$

The variable ${\gamma }_{n}$ denotes the gain by which the execution noise ${v}_{n}$ is transferred to the error signal driving adaptation. This gain differs between closed-loop trials and error-clamp trials and is directly related to the noise-compensation gain (0 ≤ $q$  ≤ 1), i.e., the fraction of the execution noise that is accounted for in the expected feedback signal (${\widehat{h}}_{n}$).

## Research questions

The current study focusses on the identification of the variance parameters in visuomotor adaptation, a problem that is of major interest also for the analysis of movement pathologies involving increased motor variability (e.g., cerebellar diseases). Before addressing such advanced questions, it is essential to develop a model suitable to describe motor variability observed under varying feedback conditions in healthy subjects. Therefore, we investigated here whether trial-to-trial variability of the pointing direction during complex adaptation experiments involving error-clamp and closed-loop conditions can be explained similarly as during simple repetitive pointing by a superposition of constant components of execution noise and planning noise or whether more complex mechanisms are involved. This is known to occur in motor skill acquisition where motor variability increases during the early training phase (Cohen and Sternad 2009). Also in motor adaptation, it has been suggested that, similar to reinforcement learning, trial-to-trial motor variability may also reflect explorative behavior that supports learning (Dhawale et al. 2017). This notion has been supported by the observation that motor variability improves error-based force-field adaptation (Wu et al. 2014). From that point of view, one could also expect an increase of motor variability during the early phase of visuomotor adaptation. A related question is whether increased motor variability affects visuomotor adaptation.

To our knowledge, these hypotheses have not been tested explicitly so far. Therefore, we developed two different models that could explain such an early increase of motor variability and test their explanatory power when fitted to experimental data. Both models extend the assumption of noise sources with constant variances by additional signal-dependent noise, i.e., white Gaussian noise with unity variance multiplied by an internally accessible signal and scaled by a constant coefficient of variation ($\kappa$). The first model implements a signal-dependent planning noise, which increases with the magnitude of the adaptive change. We assumed that such a strategic increase of explorative planning is most plausible for the fast adaptive process of the classical two-rate model since the fast process contributes mostly during the early training phase. Signal-dependent fast planning noise would therefore predict increased variability during the early training phase. The second model implements an execution noise that increases proportionally to the feedback error in the previous trial. This model would also predict an increase of the noise during the early adaptation phase when the feedback error is large. Both models differ in their prediction of how temporally increased planning or execution variance affects the inter-trial autocorrelation. Increased planning variance causes increased autocorrelation since it propagates positively to the next trial, whereas increased execution variability predicts the opposite. We will test these predictions here.

To compare these different noise models with experimental data, it is essential to correctly estimate not only the parameters determining the adaptation dynamics, i.e., the expected temporal evolution of the mean pointing direction for a given visuomotor distortion, but also the involved variance parameters. Our goal is to develop a maximum likelihood estimation method for all parameters of the system depicted in Fig. 1 observed under an arbitrary sequence of trial types involving closed-loop conditions and error-clamp conditions when the visual feedback is manipulated by the experimenter to become independent of the pointing direction.

The application of this method will not only allow the modeling of the variance/covariance structure of inter-trial noise in visuomotor adaptation to be refined, but it will also permit estimates of adaptation dynamics and variance parameters in individual subjects to be obtained with optimized precision. The best of the considered variance models will then be used to assess baseline data of the distribution of the estimated parameters across a healthy population.

### Previous approaches of model identification and their problems in estimating the variance/covariance structure of sensorimotor adaptation

In the past, methods of system identification were applied to identify the learning parameters (error sensitivities and retention rates) of linear adaptation models under closed-loop conditions. Especially the so-called direct approach to closed-loop system identification (Ljung 1997) has been applied for model identification in sensorimotor learning (Cheng and Sabes 2006; Tanaka et al. 2012; Albert and Shadmehr 2017). This approach treats the sequence of measured feedback errors (${\left\{e\right\}}_{0}^{N-2}$) as a deterministic input to the linear adaptation dynamics and the sequence of relative pointing directions $\left\{ y \right\}_{0}^{N - 1}$ as its output:

3a $$ \underline {x}_{n + 1} = \user2{A }\underline {x}_{n} + \underline {b} e_{n} + \underline {w}_{n} $$

3b $$ y_{n} = \underline {c}^{T} \underline {x}_{n} + v_{n} . $$

These equations express the input/output relation of the adaptive system and ignore the fact that, under closed-loop conditions, the error is directly related to the system states (Eq. A10). Under the assumption that both the input ${e}_{n}$ and output ${y}_{n}$ are available to the observer, the direct approach identifies the parameters of Eq. (3a/b) rather than those of Eq. (1a/b). This is much easier because the noise terms of the update (Eq. 3a) and output (Eq. 3b) are independent of each other (Eq. 2), whereas this is not the case in the closed-loop formulation (Eq. 1a/b). This independence is a necessary precondition for the use of the standard Kalman observer (Kalman 1960). The details of the standard Kalman filter are described in “Appendix A2.” For the direct approach, it is of major interest that the Kalman filter provides a highly efficient algorithm to compute the sequence of likelihoods $L\left(\left.{y}_{n}\right|{\left\{y\right\}}_{0}^{n-1}, \underline{{\vartheta }}\right)$ conditioned on the previous output observations ${\left\{y\right\}}_{0}^{n-1}$ and the system parameters $\underline{\vartheta }=\left[{\underline{\overline{x}}}_{0},\boldsymbol{ }{\varvec{A}}, \underline{b}, {\sigma }^{2}, {\varvec{W}}, {{\varvec{\Sigma}}}_{0}\right]$. By multiplying these conditional likelihoods (see Eq. A24), one obtains the likelihood of the entire output sequence (0 ≤ *n* < *N*).

4 $$ L\left( {\left. {\left\{ y \right\}_{0}^{N - 1} } \right|, \underline {\vartheta } } \right) = {\text{L}}\left( {\left. {y_{0} } \right| \underline {\vartheta } } \right) \mathop \prod \limits_{n = 1}^{N - 1} L\left( {\left. {y_{n} } \right|\left\{ y \right\}_{0}^{n - 1} , \underline {\vartheta } } \right). $$

This likelihood can then be maximized for optimal estimation of the system’s filter coefficients ${\varvec{A}}$ and $\underline{b}$. Notably, the likelihood maximized with this approach is conditioned on the observed sequence of feedback errors (${\left\{e\right\}}_{0}^{N-2}$) and does not reflect the unconditional likelihood of the observed response with respect to the set of all possible responses for a given visuomotor distortion ${\left\{u\right\}}_{0}^{N-2}$ and fixed system parameters ($\underline{\vartheta }$). For estimating the filter coefficients (${\varvec{A}},\boldsymbol{ }\underline{b}$), this is appropriate because maximizing the likelihood (Eq. 4) provides the minimum variance estimate for ${\varvec{A}},\boldsymbol{ }\underline{b}$ without bias.

In contrast, for estimating the variance parameters (${\sigma }^{2}, {\varvec{W}}$), the direct approach to closed-loop system identification induces systematic biases. This becomes obvious when considering the system in the absence of planning noise (${\varvec{W}}=0$). In this case, the residual error ${{\left\{r\right\}}_{0}^{N-1}:=\left\{y-\overline{y}\right\}}_{0}^{N-1}$ of the system with deterministic input ${\left\{e\right\}}_{0}^{N-2}$ is white Gaussian noise: ${r}_{n}\sim N\left(0, {\sigma }^{2}\right)$. In contrast, in the closed-loop system, the absence of planning noise will not lead to a white residual error, because the execution noise ${v}_{n}$ will not only contaminate the output ${y}_{n}$ but will also transfer with a negative gain to the state ${\underline{x}}_{n+1}$ and thereby also to the output ${y}_{n+1}$ (Eq. 1a). Consequently, the residuals ${r}_{n}$ and ${r}_{n+1}$ will negatively correlate with each other (van Beers 2009). Estimating the variance parameters (${\sigma }^{2}, {\varvec{W}}$) with the direct approach to closed-loop system identification causes the systematic error that the execution noise transferred to the internal memory states via feedback will be misattributed to planning noise. A second fundamental drawback of this method is that it does not account for the fact that the distribution of the observed residual (${\left\{r\right\}}_{0}^{N-1}$) depends on whether the control loop is opened or closed, even with constant variance parameters (${\sigma }^{2}, {\varvec{W}}$). This is because the recursive transfer of both the execution noise and the planning noise onto the observed output ${y}_{n}$ differs between the error-clamp and closed-loop conditions as shown in Eq. A16a–f. The direct approach (Eq. 3) cannot correctly identify the variance parameters in the closed-loop condition because it treats the input/output relation of the systems inside of the control loop as if this relation had been observed under open-loop conditions. Therefore, it will attribute all differences of the output and state variances between the error-clamp and closed-loop conditions to differences of the variance parameters (${\sigma }^{2}, {\varvec{W}}$). This is not correct because differences of the output variance occur between open- and closed-loop conditions also at constant ${\sigma }^{2}$ and ${\varvec{W}}$. Therefore, the classical direct approach to closed-loop system identification is not suitable for estimating the variance parameters in experimental designs in which error-clamp and closed-loop conditions alternate such as in the classical experiments demonstrating the rebound effect.

### Approach of the current study

Since the direct approach to closed-loop system identification, as described in the previous paragraph, does not allow the variance parameters in complex paradigms to be estimated correctly, we used the closed-loop formulation (Eq. 1a/b) throughout this study for model simulation, analytical computation of the expected model output and its variance/covariance structure, as well as for maximum-likelihood estimates of the model parameters. Even when the noise signals (${v}_{n}$, ${\underline{w}}_{n}$) were modeled as stationary processes, the model predicts that the variance and the autocovariance of the model output are not stationary as a consequence of opening the loop during the error-clamp trials. To test these predictions, we evaluated the empiric within-subject, trial-to-trial variance/autocovariance of the relative pointing direction (${y}_{n}$) within a window moving along the trial sequence. The model prediction for these measures and their changes across the adaptation experiment was computed based on the full covariance matrix ${\varvec{Y}}$ of the output vector of $\underline{y}={\left\{y\right\}}_{0}^{N-1}$ of the system defined in Eq. (1a/b). The model predictions of the mean output $\underline{\overline{y}}$, the covariance matrix ${\varvec{Y}}$, and their dependence on the model parameters $\underline{\vartheta }=\left[{\underline{\overline{x}}}_{0},\boldsymbol{ }{\varvec{A}}, \underline{b}, {\sigma }^{2}, {\varvec{W}}, {{\varvec{\Sigma}}}_{0}\right]$ are derived in “Appendix A3.” In principle, $\underline{\overline{y}}\left(\underline{\vartheta }\right)$ and ${\varvec{Y}}\left(\underline{\vartheta }\right)$ could be used to compute the likelihood $L\left(\left.\underline{y}\right|\underline{\vartheta }\right)$ of the observation for a given parameter set $\underline{\vartheta }$ and thereby for implementing a maximum-likelihood estimator for the parameters $\underline{\vartheta }$ (Eq. A30). However, this method is computationally costly because it involves numerical inversion of the large matrix ${\varvec{Y}}$, which has the square dimension of the numbers of trials (220 in our experiment). This is practically important because numerical maximization of the likelihood requires $L\left(\left.\underline{y}\right|\underline{\vartheta }\right)$ to be evaluated very often for each parameter fit. We also wanted to obtain an approximation of the expected within-subject distribution of the fitted parameters by parametric bootstrapping. For that purpose, it is necessary to first simulate many model responses for a given parameter set $\underline{\vartheta }$ and then to repeat the fitting procedure on each of those. In such a procedure, the numerical efficiency in computing the likelihood $L\left(\left.\underline{y}\right|\underline{\vartheta }\right)$ is highly important.

To provide an efficient method for computing the likelihood $L\left(\left.\underline{y}\right|\underline{\vartheta }\right)$ according to Eq. 4, we developed a generalized version of the standard Kalman observer, which allows the series of conditional likelihoods (the right side of Eq. 4) for the general closed-loop formulation of the system (Eq. 1a/b) to be computed and does not rely on the assumption of the independence of variance components of the update equation and the output equation as the standard Kalman observer does. The details of this generalized Kalman observer are presented in “Appendix A4.”

A further advantage of the method we propose here is the possibility to correctly deal with missing observations that cause a problem in the traditional “direct approach to closed-loop system identification” because the Kalman filter can deal with missing observations only if its deterministic input is known. Consequently, treating the error signal as deterministic input causes the problem that the error depends on the missing observations. In contrast, in our approach, the deterministic input to the system is not the error but the visual distortion, which is always known, even for trials with missing observations. The technical details of how our algorithm deals with missing observations are explained in “Appendices A3 and A4.”

We applied this new parameter estimation method to experimental data acquired with healthy volunteers performing two different variants of a manual reach adaptation experiment with visuomotor rotation. To achieve a more solid empirical basis, we included subjects in a wide age range to test for potential age effects on adaptation dynamics and on inter-trial variability. Pointing to a visual target was performed without vision of the hand under visual feedback of a cursor movement. The training protocol involved a block-wise sequence of baseline-, training-, washout-, and error-clamp trials where the first three blocks were performed under closed-loop conditions (cursor direction controlled by the subject). This training protocol was applied because it involves the rebound effect, which depends on the difference in the retention rates between fast and slow processes. Therefore, this protocol is most suitable to estimate the adaptation dynamics of both processes simultaneously in individual subjects. In the final error-clamp trials, the cursor movement was directed straight to the target (independent of the movement direction of the hand). To quantify the adaptive change, we evaluated for each trial the relative initial pointing direction (see “Appendix A1,” definition of ${d}_{n}$ and ${y}_{n}$) expressed with respect to its (target-specific) value during the non-adapted state (baseline). The two variants of the adaptation tasks differed in the perturbation schedule. The first introduced the visuomotor distortion gradually and did not expose the subjects to very large errors. In the second experiment, a 45 deg rotation was introduced abruptly and provoked large errors during the early adaptation phase. The comparison between abrupt and gradual training protocols was done because differences in the adaptation dynamics (error sensitivities and retention rates) between these protocols would point either to nonlinear effects of error size (Criscimagna-Hemminger et al. 2010) or to a non-stationarity of the system (Turnham et al. 2012; Herzfeld et al. 2014).

## Methods

### Subjects

Forty-nine healthy subjects were examined partly at the Centre for Vision Research, York University, Toronto, CA, and partly at the University Hospital LMU, Munich, GE. The experimental setup, the protocol, and the task were identical at both locations. The used hardware differed in only minor details. The age distribution involved two age groups with 23 subjects below 30 years (mean ± sd = 20.8 ± 1.4 years) and 20 above 50 years (62.5 ± 8.4 years). Only 6 subjects were between 30 and 50 years old. All subjects performed the task with the dominant hand (Oldfield 1971). All except two subjects were right dominant. None of them had any history of movement disorders or neurological disease.

### Apparatus and setup

**Fig. 2:**
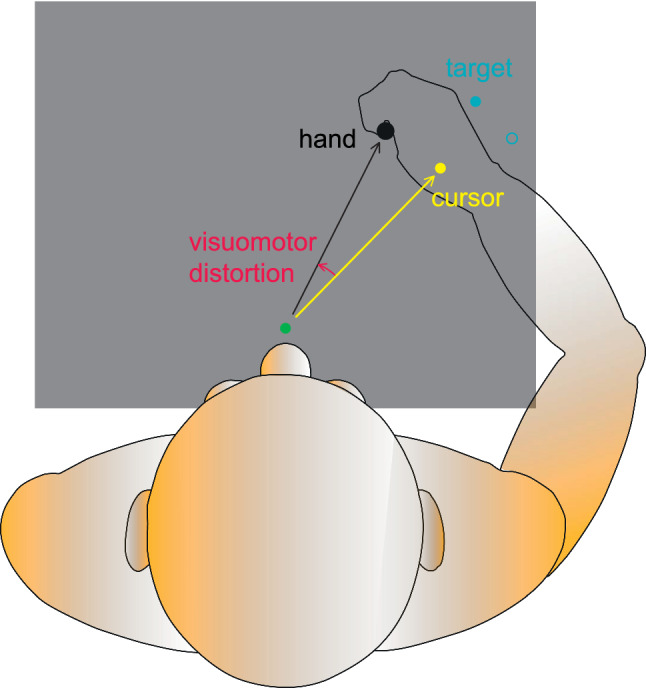
Visuomotor reach training task with rotated cursor (yellow circle). Reach targets (blue circles) were located at ± 25 and ± 35 deg from the midline. Subjects point toward the target without visual feedback of the hand. In each training block, only the two targets on the one side of the midline were shown. The distance of the cursor from the start position (green circle) was always identical to the distance of the pen from the starting point. Visuomotor distortions were induced by rotating the yellow cursor against the pointing direction of the hand around the start position (color figure online)

The experiment setup was in a semi-dark room, with a height-adjustable chair so that the subjects could sit comfortably while facing the apparatus. Subjects performed pointing movements without direct visual feedback of the hand. Hand movements were recorded with a writing tablet (WACOM Cintiq 21UX, width × height = 43.2 cm × 32.4 cm). Vision of the hand and of the arm was prevented by a reflective surface mounted horizontally and vertically centered between the surface of the writing tablet and an LCD-screen (HPL2245wg, 22″, 60 Hz) oriented downward. Subjects viewed the image on the monitor by viewing from above to a reflective surface. The reflecting surface was parallel to both monitor and tablet, so that the virtual images of the targets appeared on the plane of the writing tablet. The starting position of the movements was indicated by a green circle (diameter: 1 cm) horizontally centered and at about 15 cm from the subject (Fig. 2). The target was indicated by a blue circle (diameter: 1 cm) and was located at ± 25 or ± 35 deg to the right or to the left of the midline and at a distance of 12 cm from the starting position. Visual feedback of the hand movement was provided only by a cursor (yellow circle, diameter: 1 cm) the distance of which from the (virtual) starting point was always the same as that of the pen. Artificial distortions of the visual feedback were induced by rotating the cursor around the start position. The visuomotor rotation angle ($r$) was specified with respect to the pointing direction (defined as the interconnecting line between pen and the start position). Under *closed-loop conditions*, the visual feedback of the hand direction was directly controlled by the subject’s action since the direction of the cursor was at any time identical to the sum of the visuomotor rotation and the direction of the hand. In contrast, under *error-clamp conditions*, the cursor always moved on the straight line between the starting point and the target. The distances of the hand and of the cursor from the starting point were still identical. Thus, no visual feedback of pointing direction was available. The cursor movement direction became independent of the subject’s action. Therefore, in the context of the model (Fig. 1), the feedback loop is opened during error-clamp condition.

The pen position was acquired by a custom C-program that communicated with the tablet driver, which provided event-based position signals with variable sampling intervals. The acquisition process transferred these data online into a shared memory buffer. In this way, the MATLAB process controlling the graphics and running synchronized with the 60-Hz frame rate of the graphics card could access the actual pen position from the shared memory even though the recording of the tablet signals and the graphics output were running asynchronously. During the hand motions, the average sampling rate of the pen position signal was 136 Hz.

### Task and procedure

At the beginning of each trial, subjects moved the cursor onto the starting point and waited until the target appeared. They were instructed to move the cursor immediately after target onset toward the target. During the practice trials, they learned by verbal instruction from the experimenter to achieve three features of the cursor movements: (1) the cursor should move in a straight line to the target, (2) the movement duration should be about one second, and (3) the movements should be initiated with a clear rapid acceleration. Care was taken that these three movement features were learned in practice trials under closed-loop conditions with zero distortion before the start of the experiment. Participants were also informed that at some point during the experiment the cursor might move in an unexpected way and that their task was to maintain or restore these three features of the cursor movements as well as possible. These instructions were crucial to provide the subjects with a clear adaptation goal and to ensure that the initial movement direction could always be clearly evaluated.

The trial started with the appearance of the blue target and the simultaneous disappearance of the green starting point, which provided the go-signal for the movement. The target disappeared when the cursor stopped at the target. Immediately afterward, the green starting point reappeared, and the subject had to move the cursor back to the starting position. The next trial started when the cursor stopped at the starting point. The stopping criterion for the cursor was that it reached a distance of less than 0.5 cm from the target or the starting point at a velocity of less than 0.5 cm/s. During closed-loop trials, the cursor was permanently visible with the same visuomotor rotation. During error-clamp trials, the cursor disappeared at the end of the outward movement simultaneously with the target. The return to the starting point was performed without visual feedback until the cursor reappeared when the y-component of the distance between the pen and the start position became smaller than 2 cm.

### Experimental design

After the practice trials, the experiment started with the recording of the baseline pointing directions for each target. Subjects performed 40 trials under closed-loop conditions with zero visuomotor rotation and 40 trials under error-clamp conditions. During these baseline recordings, all four target positions were presented in pseudorandom order with balanced counts (10 presentations per target and condition).

After the baseline recordings, two blocks with 220 trials each followed. Each of these blocks was composed of an additional 40 closed-loop trials with zero visuomotor rotation, followed by a training section with 120 trials under closed-loop conditions with nonzero visuomotor rotation, 20 closed-loop trials with zero rotation (washout) and finally 40 error-clamp trials. During these 220 trials, only two of the four target positions, either on the right or on the left, were presented in randomized and balanced order. The two targets directions differed by 10 deg and occurred both with the same frequency. The randomization implied that the frequencies of the inter-presentation intervals were similar for each target and that the probability of increasing intervals decreased in an exponential fashion. Intervals 1 and 2 accounted for about 75% of all cases. The two blocks were separated by a short pause of a few minutes.

The two blocks differed only with respect to the learning sections. In the abrupt training, a visuomotor rotation of 45 deg was applied for all 120 trials of the learning section. In the gradual training, the visuomotor rotation increased during the first 60 trials of the learning section linearly from 0 to 45 deg (rate: 0.75 deg per trial) and remained constant at 45 deg for the remaining 60 trials of the learning section. Each subject performed one of four possible experimental protocols in which the first block comprised either an abrupt or a gradual training section with visuomotor rotation either to the right or to the left. Only the two targets on the left side (target directions − 25, − 35 deg) were presented during blocks with clockwise visuomotor rotation, and only the targets on the right side (+ 25, + 35 deg) during blocks with counterclockwise visuomotor rotation.

### Data analysis

For each trial, the outward movement was automatically detected based on the largest peak of the tangential hand velocity. The start and the end of the movement were defined as the time when the hand tangential velocity increased above or fell below 10% of the peak velocity. The initial movement direction was defined as the direction of the line connecting the start point of the movement with the intersection of the movement trajectory and a circle with a radius of 3 cm around the start point. In our data set, the first 3 cm of the trajectory was completed after 224 ± 53 ms, which is shorter than the reaction time (275 ms) of manual pointing movements toward (non-cued) visual targets (Barthélémy and Boulinguez 2002). Therefore, this initial movement direction was taken as an estimate of the movement direction of the feedforward component of the motor command and is called the *pointing direction* (${d}_{n}$) hereafter for brevity. The pointing direction was defined with respect to the straight-ahead direction. Positive values indicate rightward directions. The movement was marked as invalid if the peak tangential velocity stayed below 8 cm/s, if the distance between the green start marker and the start point of the movement was larger than 2.5 cm, or if the distance between the green start marker and the movement end point was smaller than 6 cm. From the pointing direction of each movement, the relative pointing direction (${y}_{n}$) was computed by subtracting the mean pointing direction averaged across the 10 error-clamp trials of the baseline recording with the same target position. The movement *n* was also marked as invalid if the relative pointing direction ${y}_{n}$ was classified as an outlier within the sample ${\left.y\right|}_{n-4}^{n+4}$. The outlier criterion was fulfilled if the distance of ${y}_{n}$ from the median was larger than 4 times the median-quartile distance. (For normal distributions, this criterion corresponds to an outlier fraction of 3%.) On average, 9% of the movements were marked as invalid.

The parameters of the two-rate model of Smith et al. (2006) were fitted to the observed relative pointing directions by maximizing the likelihood of the observed relative pointing directions. These fits were performed separately for each subject and for each training condition (gradual/abrupt). The details of this procedure are described in “Appendices 4/5/6.” In all models, the two components of the planning noise were assumed to be independent

5 $$ {\varvec{W}} = \left[ {\begin{array}{*{20}c} {w_{ss}^{2} } & 0 \\ 0 & {w_{ff}^{2} } \\ \end{array} } \right]. $$

The investigated models included not only constant variances of execution noise (${\sigma }^{2}$) and planning noise (${\varvec{W}}$) but also additional signal-dependent noise components characterized by their coefficients of variation (planning noise: $\underline{\kappa }=\left[{\kappa }_{s}; {\kappa }_{f}\right]$, execution noise: ${\kappa }_{e}$). The mathematical details of these signal-dependent noise components are described in “Appendix A5.” Model extensions with signal-dependent execution noise were previously proposed (Albert and Shadmehr 2017; Cheng and Sabes 2006; Harris and Wolpert 1998, 2006) but not modeled quantitatively to explain trial-to-trial variability in visuomotor adaptation. A possible reason is that signal-dependent execution noise was primarily introduced to describe the increase of force variability with increasing mean muscle force, whereas visuomotor adaptation does not adapt muscle force but movement direction.

Fitting the adaptation time courses of many individuals raises the question to what extent the residuals of theses fits were due to systematic or random errors of the model. To address this question, we submitted the residuals to a standard repeated measures ANOVA with one factor (*trial number*) and computed the variance components of the two random effects (*subject* and the interaction subject*trial), as well as the variance of the fixed effect (*trial number)*. This analysis was performed with the MATLAB-function “anovan.m” (The MathWorks, Inc. Version 2017b).

In our study, we compared four different models of inter-trial noise. The first model (M1) included only constant execution noise and no planning noise. The second model (M2) included constant execution noise and constant planning noise and is identical to the one used by Albert and Shadmehr (2017). Furthermore, we extended previous variance models by two different types of noise increase, which could possibly account for the increase of motor variability that is generally observed in early motor-skill acquisition and was quantified by the so-called N-Costs (Cohen and Sternad 2009). One possibility to explain such an increase is an increase of execution noise with increasing feedback error as modeled by Eq. (A46). The strength of this error-dependent execution noise is characterized by the additional variance parameter ${\kappa }_{e}$ (M3). An alternative explanation of N-Costs is an increase of planning variance during early training stages, which is proportional to the size of the adaptive change (M4). Such a signal-dependent planning noise is characterized by two coefficients of variation $\underline{\kappa }=[{\kappa }_{s}; {\kappa }_{f}]$, one for each planning state (see Eq. A49). Such a mechanism implies that the precision of visuomotor planning decreases with the magnitude of the adaptive change. The “Appendix A5” shows that both types of signal-dependent noise can smoothly be embedded in our approach of a maximum-likelihood estimation of the closed-loop system. The tested variance models differed in the constraints imposed on these parameters as shown in Table 1.

**Table 1:** Specifications of the different variance models. Model M1 accounts only for constant execution noise; Model M2 includes constant planning noise. The Models M3 and M4 extend Model M2 by additional signal-dependent planning noise or error-dependent execution noise. All models included the 7 parameters ${x}_{s0}$, ${a}_{s}$, ${a}_{f},$ ${b}_{s}$, ${b}_{f}$, $q$, and ${\sigma }^{2}$ (see “Appendix A6”)

Model ID	Name	Execution noise		Planning noise		Number of parameters (${N}_{p}$)
		${\sigma }^{2}$	${\kappa }_{e}$	${\varvec{W}}$	$\underline{\kappa }$	
M1	Constant execution noise	> 0	${\kappa }_{e}=0$	${w}_{ss}^{2}=0$ ${w}_{ff}^{2}=0$	${\kappa }_{s}=0$ ${\kappa }_{f}=0$	7
M2	Constant planning noise	> 0	${\kappa }_{e}=0$	${w}_{ss}^{2}={w}_{ff}^{2}>0$	${\kappa }_{s}=0$ ${\kappa }_{f}=0$	8
M3	Error-dependent execution noise	> 0	${\kappa }_{e}>0$	${w}_{ss}^{2}={w}_{ff}^{2}>0$	${\kappa }_{s}=0$ ${\kappa }_{f}=0$	9
M4	Signal-dependent planning noise	> 0	${\kappa }_{e}=0$	${w}_{ss}^{2}={w}_{ff}^{2}>0$	${\kappa }_{s}=0$ ${\kappa }_{f}>0$	9

Model comparisons were performed using the Akaike information criterion (Akaike 1974) evaluated as

6 $$ AIC = 2{ }N_{p} - 2{\text{ log}}\left( {L\left( {\left. {\underline {y} } \right|, \underline {\vartheta } } \right)} \right), $$

where ${N}_{p}$ denotes the number of fitted model parameters. The difference $\Delta {AIC}_{i,k}={AIC}_{i}-{AIC}_{k}$ between two models *k* and *i* fitted to the same data set is an unbiased estimator of how much larger the expected likelihood of the observed data is under the assumptions of model *k* than under that of model *i*. Since $\Delta {AIC}_{i,k}$ measures the relative likelihood on a logarithmic scale, positive $\Delta {AIC}_{i,k}$ indicates that model *k* is preferable to model *i*.

To characterize the distribution of fitted model parameters or AIC-differences across the population, we report here in general median [interquartile range (iqr)] because none of these parameters was normally distributed. This applies in particular to the slow retention rates, which were close to the upper limit one and to the variances of the slow planning noise, which were close to zero. AIC-differences frequently showed skew and long-tailed distributions. Therefore, group comparisons between younger and elderly subjects were performed using a multivariate generalization of the two-sample Wilcoxon-Mann–Whitney test of Oja and Randles (2004). The exact version of this multivariate rank-sum test is based on the distribution of its test statistic across all possible N! permutations of the N subjects. We approximated this test by evaluating the fraction of false positives from a sample of 500,000 random permutations. Multivariate paired comparisons of the adaptation dynamics (represented by the error sensitivities and retention factors) between training conditions were performed with the Oja and Randles multivariate nonparametric sign test. Univariate group comparisons were performed with the Mann–Whitney test and paired univariate comparisons (e.g., on $\Delta AIC$) with the Wilcoxon signed-rank test.

## Results

In the following, we will first compare the different models in their ability to mimic the adaptation dynamics and the inter-trial variance/covariance structure. After identifying the best variance model, in the last three sections of the Results, it will be used to assess the distribution of the estimated parameters across our subject group. Finally, evaluation of the within-subjects precision of our estimates will show how strongly it affects the between-subjects distribution.

**Fig. 3:**
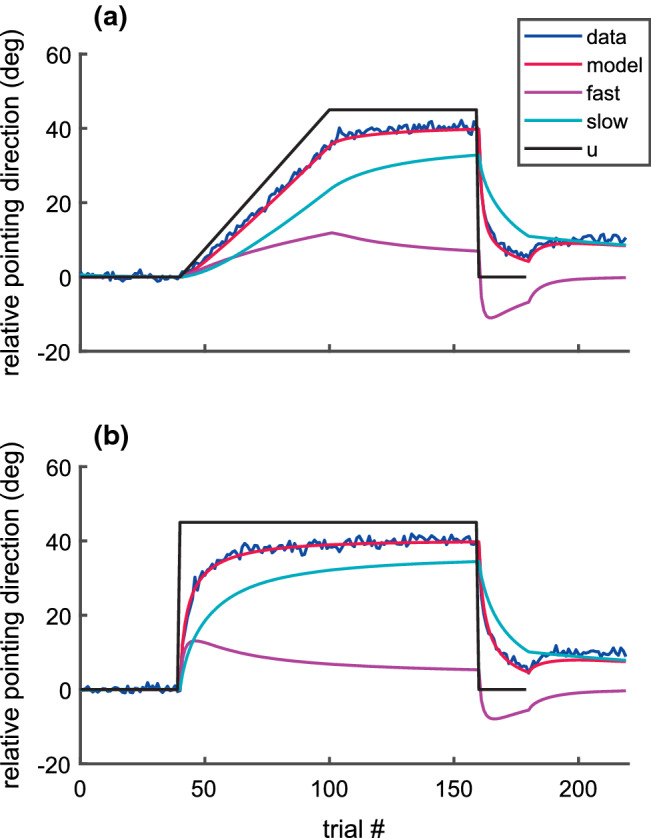
Relative pointing direction with respect to baseline. The curves show the averages of the behavioral data (blue), together with the model fit of the expected output (red, $\overline{y}$), the memory states (cyan: slow, ${\overline{x}}_{s}$, magenta: fast, ${\overline{x}}_{f}$) of the model with signal-dependent planning noise (Model M4). The model curves are means of the models fitted separately to each of the 49 subjects. The black line shows the visuomotor distortion ($u$) (color figure online)

The red line in Fig. 3 shows the mean expected relative pointing direction (${\overline{y}}_{n}$) as predicted by the model with signal-dependent planning noise (M4). The line shows the average of ${\overline{y}}_{n}$ across all individual fits. At the end of the training phase, subjects compensated for 90% (gradual: 40.52 ± 5.15 deg; abrupt: 40.29 ± 4.21 deg) of the adaptation requirement (45 deg). The difference in this final adaptive state between the training conditions (gradual/abrupt) was not significant (paired t-test: T(48) = 0.29; *p* = 0.77). As in previous studies, the model successfully explained the rebound effect in the final error-clamp trials. We also evaluated the maximum differences of the population mean of ${\overline{y}}_{n}$ predicted by model M4 and those predicted by the other models across the entire time course (0n < N). All of these three differences stayed below 1.5 deg for both gradual and abrupt training and are hardly resolvable in Fig. 3. Thus, the mean time course of the adaptation predicted by the four different models did not differ systematically. This is not surprising since the expected relative pointing directions (${\overline{y}}_{n}$) were identical across all models for any given values of their learning parameters (${\underline{\overline{x}}}_{0},\boldsymbol{ }{\varvec{A}}, \underline{b}$) and did not depend on their variance parameters ($q, {\sigma }^{2}, {\varvec{W}}, {{\varvec{\Sigma}}}_{0}, {\kappa }_{s}, {\kappa }_{f}, {\kappa }_{e}$).

The individual mean squared residuals$\left(\frac{1}{N} \sum_{n=0}^{N-1}{\left({y}_{n}-{\overline{y}}_{n}\right)}^{2}\right)$ differed systematically between models (Friedman ANOVA: ch^2^(3) = 26.95; *p* < 0.0001). The mean-squared residuals (Table 2, last column) were smaller for the model with constant execution noise only (M1: median = 29.36 deg^2^) than for the three models with more complex variance models (median = 31.85 deg^2^). The underlying reason is that the model with execution noise only predicts a sequence of residuals that resembles white noise more closely than the residuals explained by the more complex variance models. Consequently, minimizing the likelihood under the assumptions of model M1 leads to smaller residuals than under the assumptions of the other models.^1^ Figure 3 shows also that the model predicted the expected adaptation dynamics very well. To investigate to what extent the residuals of this fit were due to systematic or random errors of the model, we applied a standard repeated measures ANOVA with one factor (*trial*) to the residuals of all subjects. The estimated variance component of the random interaction (subject*trial: 32.2 deg^2^) was much larger than the variance of the fixed effect (*trial*: 0.43 deg^2^). The variance component of the random factor subject was also small (0.68 deg^2^). Thus, the great majority of the residual errors between model and data reflected random errors due to inter-trial noise, not systematic errors. This is important because it further confirms that likelihood differences between the different models do not reflect differences of the predicted mean adaptation time course but differences in the predicted noise distributions.

**Table 2:** Median [iqr] characterizing the distribution of the fitted variance parameters across the 49 individual model fits. $q$: Noise compensation gain (see Eq. A6); ${\sigma }^{2}$: constant execution variance; ${w}_{ss}^{2}$: constant planning variance of the slow memory state (${w}_{ff}^{2}={w}_{ss}^{2}$). ${\overline{w}}_{ff}^{2}$: mean of the planning variance of the fast memory state (for models without signal-dependent planning noise (M1, M2, M3), ${\overline{w}}_{ff}^{2}$ equals ${w}_{ff}^{2}$ and ${w}_{ss}^{2}$); ${\kappa }_{f}$: coefficient of variation of the fast planning noise; ${\kappa }_{e}$: coefficient of variation of the error-dependent execution noise. *msq_res*: mean square residual of the model fit

Model ID:	$q$	${\sigma }^{2}$	${w}_{ss}^{2}$	${\overline{w}}_{ff}^{2}$	${\kappa }_{f}$	${\kappa }_{e}$	*msq_res*
M1	1.00 [0.02]	27.91 [11.91]	0.00 [0.00]	0.00 [0.00]	0.00 [0.00]	0.00 [0.00]	29.36 [12.11]
M2	0.71 [0.45]	19.80 [8.73]	1.77 [2.60]	1.77 [2.60]	0.00 [0.00]	0.00 [0.00]	30.65 [15.07]
M3	0.50 [0.46]	13.81 [11.65]	1.77 [2.34]	1.77 [2.34]	0.00 [0.00]	0.22 [0.24]	32.26 [10.61]
M4	0.39 [0.33]	13.23 [10.56]	1.03 [1.89]	7.09 [5.33]	0.29 [0.28]	0.00 [0.00]	31.60 [12.56]

### Comparison between different variance models

**Fig. 4:**
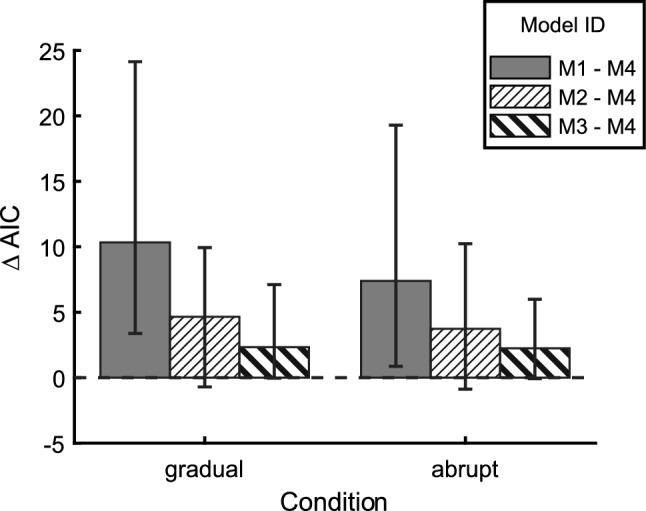
AIC-differences between the models listed in Table 1. Bars and whiskers indicate the median and the quartiles of the pairwise AIC-differences for all 49 subjects. The positive values indicate that the AIC-values of the model with signal-dependent planning noise (M4) were smaller than that of the other models

Out of all the investigated four models (see Table 1), the model with signal-dependent planning noise (M4) showed the best performance as indicated by the smallest AIC-values (Fig. 4). The median of the AIC-differences of both ${\Delta AIC}_{\mathrm{1,4}}$ (gradual: 10.3; abrupt: 7.4) and ${\Delta AIC}_{\mathrm{2,4}}$ (gradual: 4.7; abrupt: 3.7) was positive (> 2), showing that the models with constant noise sources (M1 & M2) were not or considerably less (Burnham and Anderson 2002) supported by the data when compared with the model with signal-dependent planning noise (M4). The comparison with the model with error-dependent execution noise was less clear since the median ${\Delta AIC}_{\mathrm{3,4}}$ (gradual: 2.3; abrupt: 2.2) was smaller but still positive. These results show that, during adaptation to visuomotor rotation, the assumption of constant execution noise and constant planning noise was clearly rejected by the data. The AIC analysis did not allow a clear distinction between error-dependent execution noise (M3) and signal-dependent planning noise. We also evaluated the AIC-difference ${\Delta AIC}_{\mathrm{1,2}}$ the median of which was also clearly positive (gradual: 2.8; abrupt: 2.3).

The fitted variance parameters are shown in Table 2. Compared to the model with pure constant execution noise (M1), the execution variance ${\sigma }^{2}$ decreased with increasing influence of other noise sources (M2, M3, M4). The constant component of the planning variance ${w}_{ss}^{2}$ ($={w}_{ff}^{2}=1.77$, M2) did not change when the model with constant planning and constant execution noise (M2) was extended by the error-dependent execution noise (M3). In contrast, when model 2 was extended by signal-dependent fast planning noise (M4) the constant components of planning noise ${w}_{ss}^{2}$ ($={w}_{ff}^{2}=1.03$, M4) decreased in favor of a signal-dependent fast planning noise with a coefficient of variation of ${\kappa }_{f}$=0.29. Consequently, in the best model (M4), the mean (${\overline{w}}_{ff}^{2}=7.09$ deg^2^) of the fast planning variance averaged across all trials was almost 7 times larger than the slow (constant) planning variance (${w}_{ss}^{2}$).

This shows how the relative contributions of execution noise, fast planning noise, and slow planning noise to the entire structure of inter-trial variance depend critically on the assumptions of the underlying variance model: Considering constant planning noise only (M2), previous studies (Albert and Shadmehr 2017) did not observe evidence for differences between slow and fast planning noise. In contrast, including signal-dependent planning noise, we showed that fast planning noise was much larger, but this difference was specific to the signal-dependent components and not the constant components of planning noise. This difference seems to be implied by the fact that all our three models including planning noise (M2, M3, M4) were fitted under the constraints ${w}_{ss}^{2}={w}_{ff}^{2}$ and ${\kappa }_{s}=0$. However, these constraints were not arbitrary but resulted from the analysis of two further models:

The first one (M5) was identical to the model with constant planning noise (M2) but allowed the (constant) variances for the fast and for the slow state to differ (${w}_{ss}^{2}\ne {w}_{ff}^{2}$, ${N}_{p}=9$). We found that in that case ${w}_{ff}^{2}$ (median [iqr] = 6.50 [7.48] deg^2^) was larger than ${w}_{ss}^{2}$ (1.23 [ 2.26] deg^2^). However, the AIC-difference between this less constrained model and model M2 did not differ from zero (${\Delta AIC}_{5, 2}$=0.36 [2.02]; *p* = 0.43). This analysis reproduced results of Albert and Shadmehr (2017), suggesting that the constant components of planning noise did not differ strongly between fast and slow planning processes. It also provides a first justification for our constraint ${w}_{ss}^{2}={w}_{ff}^{2}$.

The second additional model (M6) was identical with our model M4 but included unequal constant planning noise components (${w}_{ss}^{2}\ne {w}_{ff}^{2}$) and slow signal-dependent planning noise ${\kappa }_{s}>0$ (${N}_{p}=11$). In this model, the paired difference (1.52 [4.16] deg^2^) between the slow (${w}_{ff}^{2}$=2.59 [5.11] deg^2^) and the fast (${w}_{ss}^{2}$=0.63 [1.54] deg^2^) constant planning variance was smaller than in the previous model (M5), and the coefficient of variation of the slow planning noise was very small (${\kappa }_{s}$= 0.03 [ 0.05]). Model comparison showed that model M6 had even larger AIC-values than model M4 (${\Delta AIC}_{\mathrm{4,6}}$= − 2.87 [3.39]). Therefore, omitting the two constraints ${w}_{ss}^{2}={w}_{ff}^{2}$ and ${\kappa }_{s}=0$ did not improve the performance. These results suggest that a signal-dependent variance of the fast planning process is the most efficient of the considered extensions of previous variance models.

### Changes of the inter-trial variance and autocovariance of the pointing direction during adaptation

**Fig. 5:**
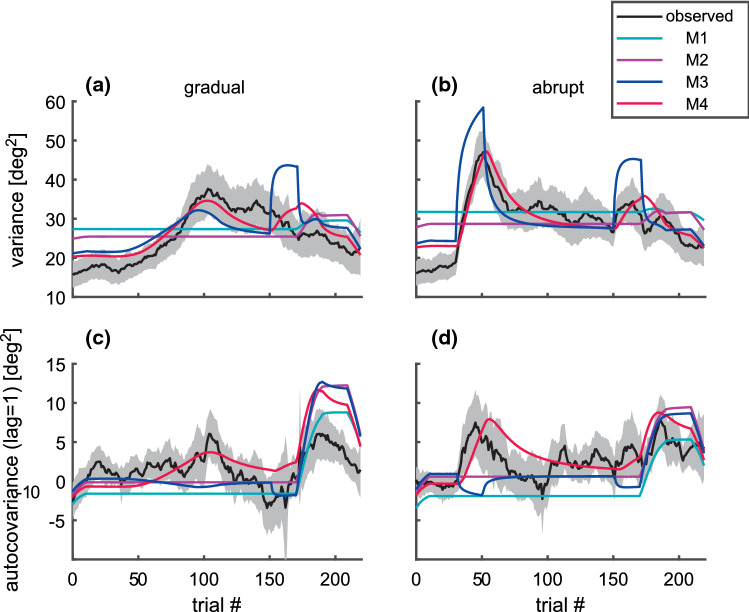
Temporal evolution of the trial-to-trial variability of the residual relative pointing direction. Lines show the variance (**a**, **b**) and the autocovariance (lag one) (**c**, **d**) of the residual evaluated in a running window of length N = 21 centered around the trial number (x-axis) for the gradual (**a**, **c**) and the abrupt (**b**, **d**) training blocks. The measured data (black) and the model predictions (colored) were evaluated separately in individuals (Eq. A25) and then averaged across subjects. The gray shaded area shows the 95% confidence interval of the observed mean (black). The models with constant noise sources only (model M1, M2) do not explain the increase of the variance during the training period. Signal-dependent planning noise (model M4) matches the observed data better than error-dependent execution noise (model M3)

The model comparison based on AIC-differences revealed that the inter-trial variability of the pointing direction was best described by constant execution variance, signal-dependent fast planning variance, and constant slow planning variance (M4). Even though each of these three noise sources was modeled as white noise, their recursive processing in the closed-loop system (Fig. 1) results in a non-white and time-dependent noise of the observed pointing direction. To understand better why model M4 provided the best match to the probability distribution of the observed inter-trial noise, we estimated the time course of its inter-trial variance (${var\_r}_{n}$) and autocovariance (${acov\_r}_{n}(\Delta =1)$) directly from the observed residuals. These two parameters were estimated in a moving window of length 21 centered around the trial *n* and compared with the expectation predicted by the different models (see “Appendix A3”). The results are shown in Fig. 5. During the closed-loop condition, the model with constant planning noise (M2, magenta) predicts small positive autocovariance (Fig. 5c, d) and fits the observed data in that respect better than the negative autocovariance predicted by the model without planning noise (M1, cyan). But both of these models with constant noise sources only do not explain the prominent temporal changes of the noise during the training phase (Fig. 5 a, b, d). The measured data show a slow increase of the variance with progressing gradual training (Fig. 5a) and a fast increase of the variance and the autocovariance immediately at the beginning of the abrupt training (at trial 40, Fig. 5b, d). In approximate agreement with the data, both model M4 and M3 predicted a gradual variance increase of the gradual training (Fig. 5a) and an abrupt variance increase of the abrupt training (Fig. 5b). The later was overestimated by model M3 (blue line in Fig. 5b, trial40). Both models differed in their predictions of the change of the autocovariance at the onset of the abrupt training onset. The model with signal-dependent planning noise (M4) correctly explains the sudden increase of the autocovariance (Fig. 5d, trial40), whereas the model with error-dependent execution noise (M3) makes here the opposite, incorrect prediction. This is because planning noise (${\underline{w}}_{n}$) at trial *n* enters into the pointing direction ${y}_{n+1}$ of the following trial with a positive sign and execution noise (${v}_{n}$) with a negative sign (Fig. 1). Model M4 also outperforms model M3 in that it does not predict a strong variance increase at the beginning of the washout phase (at trial 160, Fig. 5a, b). This difference between the models reflect the fact that the increase of the feedback error is larger than that of the fast state. This can be seen, for example, in the gradual training (Fig. 3a) where the absolute feedback error changed by 130% from 5.24 deg at trial 160–12.05 deg at trial 165, whereas the fast planning state changed only by 57% (from 6.98 to 10.98 deg) in the same interval.

Opening the loop (at trial 180) induced an increase of the observed autocovariance in the gradual training (Fig. 5c) that was less pronounced as predicted by all models. The differences of the observed time courses (black) between the training conditions (gradual/abrupt) were explained by the signal-dependent planning noise of model M4, even though its variance parameters ($q$, ${\sigma }^{2}$, ${w}_{ss}^{2}$, ${\kappa }_{f}$) did not show such a difference (see above). Because model M4 was the best one considered, all population statistics of the fitted model parameters reported in the following will refer to the parameters of model M4.

### The distribution of the noise parameters across subjects

**Fig. 6:**
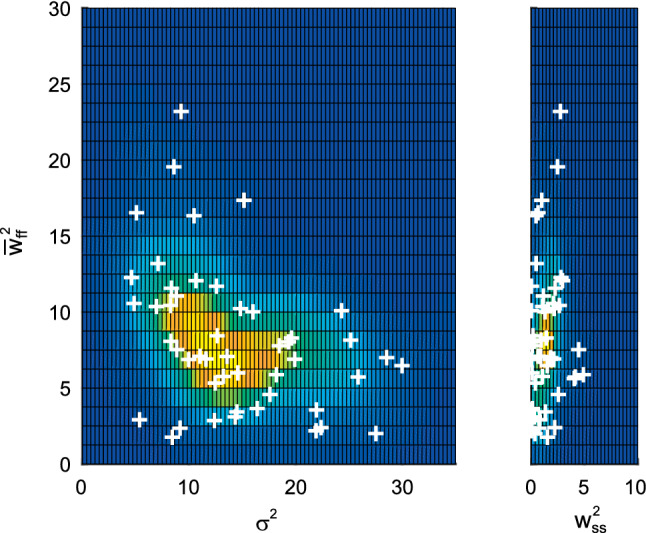
The distribution of the variance parameters of the model with signal-dependent fast planning noise (M4) across the 49 subjects (crosses). Data show the average between gradual and abrupt training conditions. According to this model, the trial-to-trial variability of each subject is characterized by three variances. The execution variance (${\sigma }^{2}$) and the average of the signal-dependent fast planning noise across all trials (${\overline{w}}_{ff}^{2}$) have similar sizes, whereas the constant slow planning variance (${w}_{ss}^{2}$) is clearly smaller. The heat map shows a smoothed density estimation

The distribution of the noise parameters (M4) is shown in Fig. 6. The execution noise correlated negatively with the mean fast planning variance (Spearman’s ρ(${\sigma }^{2}$, ${\overline{w}}_{ff}^{2}$) =  − 0.42; *p* < 0.01) and with the coefficient of variation of the fast planning noise (ρ(${\sigma }^{2}$, ${\kappa }_{f}$) =  − 0.36; *p* < 0.02). Execution noise and constant planning noise did not correlate significantly with each other. The fitted noise parameters ($q$, ${\sigma }^{2}$, ${w}_{ss}^{2}$, ${\kappa }_{f}$) of model M4 did not differ between the training conditions (gradual/abrupt) (Oja & Randles multivariate sign test: chi2(4) = 7.71; *p* = 0.10). None of the four univariate comparisons of these variance parameters between gradual and abrupt training was significant (Wilcoxon signed-rank: *p* > 0.09). To test for potential effects of age on the trial-to-trial variability, we compared these four variance parameters between subjects younger than 30 (N = 23) and older than 50 (N = 20). Differences of $\left[q, {\sigma }^{2}, {w}_{ss}^{2}, {\kappa }_{f}\right]$ between the age groups did not reach significance (Oja & Randles multivariate rank sum test: *p* > 0.1) for any training condition (gradual/abrupt).

### The distribution of error sensitivities and retention rates across subjects

Table 3 shows the median and interquartile ranges of the distribution of error sensitivities and retention rates across all subjects (M4). The distribution of $\left[{a}_{s}, {a}_{f}, {b}_{s}, {b}_{f}\right]$ differed significantly between the training conditions (Oja & Randles multivariate sign test: chi2(4) = 15.06; *p* = 0.0046). This effect was due to a highly significant (Wilcoxon signed-rank test: *p* = 0.0001) decrease of the fast error sensitivity in the abrupt (${b}_{f}$= 0.17) compared to the gradual training condition (${b}_{f}$= 0.26). The fitted learning parameters of model M4 were also compared between subjects younger than 30 (N = 23) and older than 50 (N = 20). Differences of $\left[{a}_{s}, {a}_{f}, {b}_{s}, {b}_{f}\right]$ between these age groups did not reach significance (Oja & Randles multivariate rank sum test: *p* > 0.15) for any training condition (gradual/abrupt). Together with the absence of age effects on the variance parameters (see above), this result shows that, in our subject group, age did not systematically affect adaptation dynamics or trial-to-trial variability. Therefore, pooling across all subjects for the purposes of model selection was justified. The decrease of the fast error sensitivity in the abrupt training compared to the gradual training manifests in the downward step of the fast state at the beginning of the washout period (trial 160), which was larger in the gradual (Fig. 3a, magenta) than in the stepwise training (Fig. 3b, magenta). As a consequence of this effect, the average speed of the deadaptation between trials 160 and 163 was faster in the gradual (− 9.17 [3.97] deg/trial) than in the abrupt training condition (− 7.44 [5.01] deg/trial). The paired difference (1.94 [6.03] deg/trial, N = 49) was significant (Wilcoxon signed-rank test: *p* = 0.02).

**Table 3:** Median [iqr] of the retention rates ($\left[{a}_{s}, {a}_{f}\right]$) and error sensitivities $\left[{b}_{s}, {b}_{f}\right]$ across all 49 subjects (model M4) for the gradual and the abrupt training conditions. The difference of the fast error sensitivity between the conditions (third row, column ${b}_{f}$) was negative (Wilcoxon signed-rank test; row four), indicating that the fast error sensitivity was larger in the gradual than in the abrupt condition

	${a}_{s}$	${a}_{f}$	${b}_{s}$	${b}_{f}$
Gradual	0.99 [0.02]	0.78 [0.34]	0.10 [0.06]	**0.26** [0.13]
Abrupt	0.99 [0.01]	0.72 [0.48]	0.08 [0.08]	**0.17** [0.14]
Difference	− 0.00 [0.02]	0.02 [0.37]	− 0.00 [0.09]	** − 0.10** [0.16]
*p* (signed-rank)	0.3523	0.6944	0.9722	**0.0001**

Across the population, the total adaptive change at the end of the training period (${\left.{\overline{y}}_{n}\right|}_{n=159}$) did not depend on the mean-squared residual error (Spearman’s rank correlation coefficient: gradual: $\rho =$ 0.13; *p* = 0.38; abrupt: − $\rho =$0.17; *p* = 0.25), the average of the planning variance across trials (${\overline{w}}_{ff}^{2}+{w}_{ss}^{2}, \left|\rho \right|$<0.12; *p* > 0.3), or the average of fast planning variance (${\overline{w}}_{ff}^{2}, \left|\rho \right|$<0.4; *p* > 0.7). Thus, the overall adaptation performance was not related to the trial-to-trial variability.

### Observability of the learning dynamics and variance parameters

The between-subjects variance is the sum of the variance due to real differences between individuals and the variance due to the trial-to-trial noise, which limits the precision of the parameter estimation in each subject. Therefore, we asked for the relative contribution of the within-subject variance to the between-subject variance. Based on the parameters individually fitted with the model, we simulated for each subject and for each gradual and abrupt training experiments 600 pointing sequences. The population medians and iqrs of the used learning parameters are shown in Table 3 and the variance parameters in Table 2 (model M4). Model M4 was then fitted to each of these 49 × 2 × 600 = 58,800 simulated data. The variance of the resulting model parameters across these fits reflects the estimation noise of our method. These within-subject variances of the learning parameters were pooled across all subjects and then compared with the experimentally observed between-subjects variance (Table 4). The relative contribution of estimation noise to the total between-subjects variability was considerable (between 20% for ${a}_{f}$ and 46% ${a}_{s}$). The estimation noise was also larger in the parameters of the fast adapting process (${a}_{f}, {b}_{f}$) than in the slow adapting process (${a}_{s}, {b}_{s}$). Nevertheless, the difference of the population variance between the fast and the slow processes is not explained by the inferior observability of the fast process only because the relative contribution of the estimation noise to the total between-subjects noise was even smaller in the fast than in the slow process (Table 4, rows *within/between*). Thus, the large differences of the fast learning parameters between individuals reflect behavioral differences.

**Table 4:** The variances of the retention rates (${a}_{s}, {a}_{f}$) and error sensitivities (${b}_{s}, {b}_{f}$), fitted with model M4. The variance across the population (*between variance*) is compared with the variance and across simulated pointing sequences (*within variance*) for both gradual and abrupt training conditions. The row labeled “within/between (%)” shows the relative contribution of the estimation noise to the total observed variance across the population in percent. The variances of the parameters of the fast process are larger than that of the slow process. In contrast, relative contributions of the within-subject noise were smaller in the fast than in the slow process

		${a}_{s}$	${a}_{f}$	${b}_{s}$	${b}_{f}$
Gradual	Between-variance	1.11 e-4	5.46 e-2	1.95 e-3	1.22 e-2
	Within-variance	0.45 e-4	1.05 e-2	0.95 e-3	0.46 e-2
	Within/between (%)	40.1	19.3	48.5	37.5.1
Abrupt	Between-variance	8.96 e-5	7.23 e-2	2.60 e-3	1.01 e-2
	Within-variance	4.60 e-5	1.49 e-2	0.63 e-3	0.22 e-2
	Within/between (%)	51.3 .5	20.6	24.2	22.1

The same model fits to simulated data were also used to investigate how the estimation of the variance parameters of the two-rate adaptation model of Smith et al. (2006) depends on the choice of the variance model. Of practical interest is the absolute size of the estimation errors induced by choosing variance models that do not correctly fit to the statistics of the observations. We included in this analysis also the standard regression model, which assumes white Gaussian noise on the observed pointing directions, and for which the method of least squares (LSQ) is identical with maximizing the likelihood of the observation. The standard regression is a special case of the general formulation of Eq. (A16a–f), characterized by the absence of planning noise (${\underline{w}}_{n}=\underline{\_}{0}$), and no noise transfer from the motor output to the error driving adaptation ($\gamma =0$) in closed-loop and error-clamp trials. Table 5 shows that the errors were smallest for the model M4 that fully accounted for the variance structure of the simulated data. The two models ignoring planning noise (LSQ, M1) cause errors in the estimation of the variance of the execution noise (${\sigma }^{2}$) of up to 10 deg^2^. The model assuming constant variance of execution and planning noise (M2) showed errors that were larger ($q$: 115%, ${\sigma }^{2}$: 61%, ${w}_{ss}^{2}$: 147%, ${\overline{w}}_{ff}^{2}$: 144%) than the errors of model M4. This demonstrates that the estimation of the variance parameters depends critically on the completeness of the model. Making a wrong assumption about only one parameter such as the assumed constancy of the fast planning noise (M2) affects all other variance estimates.

**Table 5:** Median absolute error of the variance parameters fitted to model simulations. For each subject and each training condition (gradual/abrupt), 600 experimental runs were simulated based on model M4 and on its parameters obtained by fitting M4 to the 49 × 2 individual experimental data. Each of these 49 × 2 × 600 simulated data sets was then fitted by four different models. *LSQ* least squares; M1, M2, M4: Maximum-likelihood fits of closed-loop models (Table 1). Shown is the median of the absolute difference between the fitted parameters and the “true” ones used for the simulation (N = 58,800). Ignoring planning noise (LSQ, M1) causes large errors in the estimation of execution noise (${\sigma }^{2}$). The absolute errors of model M2 ignoring the signal dependency of the fast planning noise were between 60 and 150% larger than those of M4

Model ID:	$q$	${\sigma }^{2}$[deg^2^]	${w}_{ss}^{2}$[deg^2^]	${\overline{w}}_{ff}^{2}$[deg^2^]	${\kappa }_{f}$
LSQ	–	9.83	–	–	–
M1	0.526	10.42	–	–	–
M2	0.369	3.85	0.663	4.22	–
M4	0.172	2.39	0.268	1.73	0.060

**Fig. 7:**
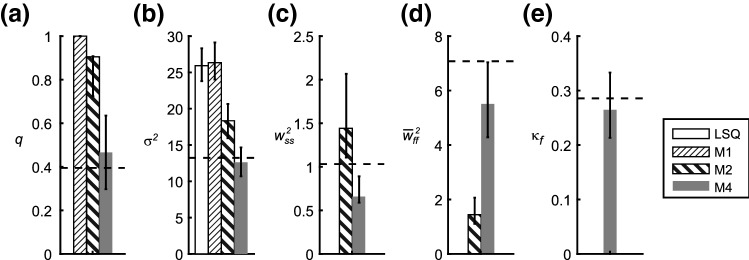
Variance parameters fitted to simulated data. The execution noise-compensation gain ($q$) and the coefficient of variation of the fast planning noise (${\kappa }_{f}$) are dimensionless, the variances of execution noise (${\sigma }^{2}$) slow (${w}_{ss}^{2}$) and fast (${\overline{w}}_{ff}^{2}$) planning states are shown in units of deg^2^. Dashed horizontal lines: median of the parameters used for simulation (see Table 2, row M4). Bars: Median (across 2 × 49 parameter sets) of the medians of the parameters fitted to the 600 simulated data sets per individual and condition (gradual/abrupt). Whiskers: the length indicates the upper and the lower median-quartile distance within the 600 model simulations with the same underlying parameter set (uncertainty of the parameter estimation due to trial-to-trial noise)

The median absolute errors shown in Table 5 include both systematic and random estimation errors. To evaluate the systematic errors, we computed the medians of the parameter estimates across the 600 simulations separately for each of the 2 × 49 data sets and compared the median of these estimates (Fig. 7, bars) with the median of the “true” variance parameters (Fig. 7, dashed horizontal lines) used to create these 49 × 2 data sets. The noise-compensation gain $q$ and the execution variance ${\sigma }^{2}$ were overestimated by model M1, M2 (Fig. 7a, b). Similar overestimation of ${\sigma }^{2}$ was also obtained by the LSQ-fit. Both models including constant planning variance (${w}_{ss}^{2}$) showed systematic differences between the estimated and the “true” value: M2 overestimated and M4 underestimated ${w}_{ss}$ (Fig. 7c). A stronger discrepancy between the models M2 and M4 occurred in the mean fast planning variance (${\overline{w}}_{ff}^{2}$), which was more underestimated by the model with constant planning variance (M2) than by model M4 (Fig. 7d). This reflects the fact that model M4 could appropriately mimic the time dependency of the fast planning noise with its coefficient of variation ${\kappa }_{f}$ of 0.29 (Fig. 7e).

The uncertainty of the parameter estimation due to the trial-to-trial variability was quantified by the median-quartile distance of the estimate distribution within the 600 fits to the simulations with the same “true” parameter set (whiskers in Fig. 7). Compared to the other models, M4 showed a smaller interquartile range of this distribution for the variance of the slow planning noise (${w}_{ss}$, Fig. 7c) only. For the variance of the execution noise, the estimation noise was similar for all models (whiskers length in Fig. 8b). This shows that smaller systematic errors rather than smaller random errors of model M4 were mainly responsible for the better performance of model M4 concerning the absolute errors (Table 5).

Fitting model M4 many times to data sets simulated with the same underlying parameter set also allowed the statistic dependence between the different variance parameters to be investigated on the level of the estimation noise. Correlating the execution noise with the mean fast planning variance across the 600 simulations within each of the 49 × 2 data sets showed a Spearman’s rank correlation coefficient with a median of ρ(${\sigma }^{2}$, ${\overline{w}}_{ff}^{2}$) =  − 0.57 [0.27] (N = 98). The median and the interquartile range of the Spearman’s correlation between execution noise and the coefficient of variation were ρ(${\sigma }^{2}$, ${\kappa }_{f}$) =  − 0.33 [0.17]). That these correlations did not differ from the ones observed across the population as reported above (ρ(${\sigma }^{2}$, ${\overline{w}}_{ff}^{2}$) =  − 0.42; ρ(${\sigma }^{2}$, ${\kappa }_{f}$) =  − 0.36) suggests that the negative correlation between execution noise and fast planning noise reflects a feature of the estimation noise rather than a feature of the parameter distribution across subjects.

## Discussion

We developed and applied a maximum-likelihood estimation method for a closed-loop linear adaptation model including two different types of signal-dependent noise. The model comparison confirmed previous results (van Beers 2009) that a combination of execution noise and planning noise accounted better for the nonnegative autocovariance (lag one) of the pointing direction (${\Delta AIC}_{\mathrm{1,2}}>0$) than execution noise alone. Extending previous studies, we showed that the assumption of constant execution noise and constant planning noise is not fulfilled during visuomotor adaptation. A model with constant execution variance, equal constant planning variance for both slow and fast adapting planning states, and additional signal-dependent fast planning noise (M4) showed smaller AIC-values (Fig. 4) and matched the time course of the variance and of the autocovariance of the within-subject (trial-to-trial) noise better than all other models (Fig. 5). Even though the model with error-dependent execution noise (M3) could not clearly be rejected in favor of signal-dependent planning noise (M4) based on AIC alone, M4 was strongly supported by the increase of the autocovariance (lag 1) that occurred simultaneously with the fast increase of the output variance (Fig. 5b, d, trial 40). Increased execution noise would have had just the opposite effect on the autocovariance.

Throughout this study, we simplified modeling by assuming that errors occurring at a particular movement direction contribute equally to the adaptation of movements of the other direction. This is an oversimplification since it is known that visuomotor adaptation of a single target direction generalizes by only about 70% to target directions that differ by 22.5 deg (Krakauer et al. 2000). In the present study, two target directions, separated by 10 deg, were presented randomly at equal probability. In this case, according to the modeling approach of Tanaka et al. (2012), ignoring the lack of generalization when the error sensitivity for the neighbored target equals only a fraction (*f* < 1) of the sensitivity for the trained target would lead to an underestimate of the error sensitivities (on the trained direction) in range of $(1+f)/2$. The estimates of the retention rates are not expected to be systematically affected by the simplification. However, our new method to compute the maximum likelihood of the observed pointing direction could easily be applied to the multi-target adaptation model of Tanaka et al. (2012). This model is a time-variant discrete linear filter with a state space, which is extended to a dimension $2M$, where *M* denotes the number of trained target directions. Therefore, the extended state-space model for multi-target adaptation is a special case that is formally covered by the time-variant formulation (Eq. A16a/b) and all further derivations of the appendices.

### Differences between gradual and abrupt training

Overall, we did not observe strong differences of the adaptation dynamics between gradual and abrupt training. The retention rates of both fast and slow mechanisms, as well as the slow error sensitivity, were identical for both training conditions. Only the fast error sensitivity was significantly smaller in abrupt than in gradual training. This effect confirms that the adaptation dynamics are not independent of the history of the presented errors as shown by previous studies. Zarahn et al. (2008) argued that such effects of metalearning can be modeled by changes of the learning parameters across the phases of an adaptation experiment. Huang and Shadmehr (2009) found in force field adaptation that retention rates measured during error-clamp trials were larger after gradual than after abrupt training. Turnham et al. (2012) observed in adaptation to visuomotor rotation that the fast adapting process showed larger error sensitivity and, to a lesser degree, a larger retention rate after gradual or random training than under control conditions. These effects were interpreted in the context of a model that predicts that the error sensitivity decreases when errors change inconsistently (Herzfeld et al. 2014).

### Maximum-likelihood estimation of variance parameters of a closed-loop system

The maximum-likelihood estimator is asymptotically bias-free and has the best efficiency of all normally distributed estimators (Fisher 1925), i.e., the smallest variance for a given number of observations. Applied to linear models with additive Gaussian noise (like the two-rate adaptation model), it has two major additional benefits that make it the generally preferred estimator: First, the estimates of the filter coefficients (i.e., the parameters determining the expectation of the model output) are bias-free not only asymptotically but even for small samples. Second, even using wrong variance models does not induce biases for the estimates of the filter coefficients. For that reason, the linear regression model (LSQ) is widely used even if its strong assumption of white noise is not strictly justified. Unfortunately, this robustness only applies for the filter coefficients, not for the variance parameters (i.e., the parameters determining the noise distribution). The best-known example is the maximum likelihood estimate of the variance in a simple constant model with additive Gaussian noise ${x}_{i}=m+{r}_{i}$ for 1iN with ${r}_{i}\sim N(0, {\sigma }^{2})$. The maximum-likelihood estimator of the variance is ${\widehat{\sigma }}^{2}=\frac{1}{N} \sum_{i=1}^{N}{\left({x}_{i}-m\right)}^{2}$, which is not bias free but has the expectation $E\left\{{\widehat{\sigma }}^{2}\right\}=\frac{N-1}{N} {\sigma }^{2}$. Similarly, our maximum likelihood estimator with the variance model that accounted fully for the variance structure of the simulated data (M4) is also not completely bias-free. Figure 7 shows that the planning noise (${w}_{ss}$, ${\overline{w}}_{ff}$) was underestimated by M4. However, Fig. 7 also shows that using a wrong variance model for estimating the variance has more severe consequences on the estimates of the variance parameters. Ignoring the signal dependency of the fast planning noise (M2) causes the noise-compensation gain $q$, the execution variance ${\sigma }^{2}$, and the slow planning variance ${w}_{ss}$ to be overestimated. The fast mean planning noise ${\overline{w}}_{ff}$ is underestimated. Overall, Fig. 7 demonstrates that our maximum likelihood estimator is able to identify the complex variance structure of these data most accurately when it is based on the correct model (M4). This is important because the observed variance and autocovariance of the behavioral data (Fig. 5, black) could not be modeled appropriately by constant execution and planning noise (M1, M2). Thus, the complexity of model M4 is required to describe the pointing variability in our experiment and must therefore also be considered in a maximum likelihood estimator to identify its variance structure. The algorithm for computing the exact likelihood of the closed-loop system, which is described in “Appendices A3/4/5,” is therefore a necessary tool for the purpose of this study.

An interesting new aspect of our variance model is that it allows an empirical estimate for the noise-compensation gain ($q$) to be obtained. Previous studies estimating planning noise (Albert and Shadmehr 2017; Zarahn et al. 2008) in visuomotor adaptation ignored the possible transfer of execution noise into the error signal ($\gamma =0$) and could therefore apply the standard Kalman filter for computing the likelihood of the output (see “Appendix A2”). One of the main problems with this assumption is that in closed-loop trials this corresponds to a compensation gain of $q=1$, whereas in error-clamp trials $\gamma =0$ corresponds to $q=0$. The difficulty to justify this assumption in experiments involving both closed-loop and error-clamp trials was one of the main motivations to develop the generalized Kalman filter for the closed-loop system (“Appendix A4”). The median of $q$ was 0.39 (Table 2, line 4), suggesting that the expected visual feedback accounts for 39% (population median) of the execution noise contaminating the actual visual feedback in the closed-loop trials.

In a previous study, Albert and Shadmehr (2017) already evaluated the estimation errors of the learning parameters (${a}_{s}$, ${a}_{f}$, ${b}_{s}$, ${b}_{f}$) in individuals and obtained similar simulation results as the ones shown in Table 4 (within variance). Thus, our extension of the underlying variance model had only marginal benefit for the estimation of the learning parameters. However, substantial benefit was not expected since in linear models, the efficiency of the coefficient estimates is relatively robust with respect to small errors of the variance model. One benefit of our method is that it allows analysis of the precision of the variance estimates obtained in a single subject (whiskers in Fig. 7, M4). Of primary interest is here not only the interquartile range of the within-subject distribution per se, but also its ratio to the interquartile range observed across the population (Table 2, line 4, [iqr]). Values of this ratio close to zero indicate that the corresponding variance estimate can be used as an individual characteristic, whereas values close to one indicate the opposite. These ratios were $q$: 1.02; ${\sigma }^{2}$: 0.38 deg^2^; ${w}_{ss}^{2}$: 0.16 deg^2^; ${\overline{w}}_{ff}^{2}$: 0.52 deg^2^; ${\kappa }_{f}$: 0.43. They show that, similar to the learning parameters (Table 4), a considerable part of the dispersion across subjects was due to the limited precision of the estimation in a single subject. Especially the noise-compensation gain $q$ proved to be not suitable for characterizing an individual, despite its small estimation bias (Fig. 8a, M4). In contrast, estimates of ${w}_{ss}^{2}$, ${\sigma }^{2}$ and, to a lesser extent also ${\kappa }_{f}$, allow inter-individual comparisons.

### Time-variant planning noise during visuomotor adaptation

On the one hand, our observation that trial-to-trial variability increased during the early part of visuomotor adaptation resembles the increase of execution variability during early motor skill learning. But on the other hand, we did not find any hint that the overall performance of visuomotor adaptation improved with increasing variance of the residual, the total planning variance, or the fast planning variance. Thus, in contrast to the reward-based motor learning task of Wu et al. (2014), we did not find any benefit of execution noise on task performance. This indicates that the execution variability may be more important in the reward processing task of Wu et al. (2014) that resembled a reinforcement learning task more closely than the visuomotor adaptation of the current study. Our finding confirms the classical two-rate model in that its expected adaptation time course does not depend on the noise level. At the same time, the current study demonstrates that the variance of the fast adapting memory state is modulated in time even though this noise was not beneficial for the adaptation task (absence of a correlation between ${\overline{w}}_{ff}^{2}$ and the total adaptive change). This is not contradictory, as increased variability also did not impair adaptation in our study even though such an impairment might be expected based on studies investigating the effect of error consistency on error sensitivity (Herzfeld et al. 2014) or studies suggesting a direct influence of the noise level on the error sensitivity due to a statistic evaluation of error relevance (Berniker and Kording 2011; Wei and Körding 2009). The absence of effects of execution or planning variance on the total adaptive change suggests that potential nonlinear contributions of trial-to-trial noise did not have any effect, either positive or negative. It seems therefore that the fast adapting planning process is linked with a mechanism of active variance control, possibly because the same planning process is also involved in reinforcement learning tasks where it is beneficial. The participation of this planning process in visuomotor adaptation does not require modification of its associated variance control because it is irrelevant in the adaptation task.

Our interpretation that the temporal modulation of the observed trial-to-trial variance was due to signal-dependent fast planning noise is supported by the finding that the increase of the trial-to-trial variance in the early adaptation phase closely followed the time course of the fast adapting planning state which increased more slowly and reached its peak later in the gradual than in the abrupt training (Fig. 3, magenta). The same variance parameters of model M4 could explain the two different time courses of variance and autocovariance in both conditions (gradual/abrupt; Fig. 5). Thus, the observed variance increase was specifically linked to the contribution of the fast adapting process. This specificity supports the notion that fast and slow components of visuomotor adaptation represent distinct and separable mechanisms. Originally, the two-rate model did not implicate a clear separability of the underlying processes (Smith et al. 2006). Later studies (McDougle et al. 2015) suggested that fast and slow adaptation mechanisms are associated with explicit and implicit motor learning. The present study contributes to this discussion in that it shows that the fast mechanism but not the slow one is involved in the control of planning variability. This is a functional distinction between the two mechanisms even though it remains unclear whether control of planning variability is an explicit or implicit process.

The current study tested the alternatives regarding whether the observed variance increase in the early phase of visuomotor adaptation was due to non-stationary (error-dependent) execution noise or non-stationary (signal-dependent) planning noise. The sudden increase of the autocovariance at the beginning of the training phase suggested that the non-stationary planning noise was responsible for the observed variance increase. We showed here that signal-dependent planning noise can explain the presented results, but it has to be noted that other mechanisms of non-stationary planning noise may provide possible alternatives. For example, planning variability may be conceived as a combination of genuine (constant) planning noise and exploration. Exploration is not needed when performance is fine and increases only with larger errors when exploration is beneficial. Such a mechanism, which can be characterized as error-dependent planning noise, was previously discussed mainly in the context of reinforcement learning. The current study cannot distinguish between such error-dependent planning noise and signal-dependent fast planning noise because both the error and the fast adaptive component show large values in the early training phase.

In summary, the modified Kalman approach developed in this study allowed the variance parameters of time-discrete adaptive systems observed under varying feedback conditions to be estimated. This method allowed modeling of the temporal changes of the trial-to-trial variance and autocovariance of the pointing direction in a complex training paradigm in visuomotor adaptation. The results showed that trial-to-trial variability increased during the early learning phase similar to motor skill learning. This increase was due to an increase of planning noise rather than execution noise and could be modeled by signal-dependent fast planning noise.

## Data Availability

The datasets generated during and/or analyzed during the current study are available from the corresponding author on reasonable request.

## Appendix

### A1: Model definition

The structure of a two-rate model of sensorimotor adaptation is shown in Fig. 1. This model is almost identical with the two-rate adaptation model proposed and applied by many previous studies (Smith et al. 2006; Criscimagna-Hemminger and Shadmehr 2008; Albert and Shadmehr 2017; Ethier et al. 2008; Zarahn et al. 2008). Figure 1 visualizes the mathematical relations between the internal memory states represented here by the two components of the two-dimensional state vector (*x*_*n*_), the observed pointing direction (*d*_*n*_), and the experimentally induced visuomotor distortion (*u*_*n*_). Earlier studies did not fully investigate the implications of this model for the variance/covariance structure of the pointing sequence (${\left\{d\right\}}_{0}^{N-1}$). In particular, the dependence of the variance of pointing direction on the trial type (closed-loop, error-clamp) was never compared with the predictions of this model. Therefore, the current study focuses on that aspect.

*n*: Trial number. The experiment is composed of *N* trials which are numbered from *n* = 0 to *n* = *N* − 1. In the following, quantities that are specific for each trial appear with the index *n*.

*d*_*n*_ (deg): Pointing direction. The pointing direction was defined as the direction of the line connecting the start point of the movement with the intersection of the movement trajectory and circle with a radius of 3 cm around the start point. The pointing direction is specified with respect to the straight-ahead direction. Positive values indicate rightward directions.

*g*_*n*_ (deg): Motor goal. The angle *g* specifies the average direction of the pointing movement in the non-adapted state. It is identical to the sum of the target direction and potential motor biases for a given target and is defined relative to the straight-ahead direction. Positive values of *g*_*n*_ indicate movements to the right. The motor goal is defined as the mean pointing direction that is adopted when the subject is fully familiarized with the pointing device in the absence of any artificial visuomotor rotation (*r*_*n*_). Therefore, the motor goal is also called the baseline pointing direction and is specific for the direction of the target presented in trial *n*. The functional dependence of the motor goal on target direction is assumed to be independent of time. Thus, the index *n* in *g*_*n*_ does not reflect temporal changes of that function but only that the target direction differed from trial to trial.

*vd*_*n*_ (deg): Visually guided pointing direction. This angle is the pointing direction that would be executed in the absence of planned motor exploration, external transient mechanical perturbations, and motor noise. All these types of noise are subsumed under the term “execution noise” (*v*_*n*_)

A1 $$ vd_{n} = d_{n} - v_{n} . $$

*v*_*n*_ (deg): Execution noise. We assume that the execution noise is distributed normally with zero-mean and variance σ^2^

A2 $$ v_{n} \sim N\left( {0,{ }\sigma^{2} } \right). $$

*r*_*n*_ (deg): Visuomotor rotation. The angle *r*_*n*_ specifies the rotation of the cursor with respect to the pointing direction of the hand. Positive *r*_*n*_ indicates clockwise rotation of the cursor. The actual visual feedback (*h*_*n*_) is the sum of the pointing direction and the visuomotor distortion

A3 $$ h_{n} = d_{n} + r_{n} . $$

The artificially introduced visuomotor rotation is the quantity that causes the systematic errors driving the motor adaptation. Clockwise visuomotor rotations induce counterclockwise changes in the visually guided pointing direction. To use the same sign convention for the driving stimulus and for the resulting adaptive change, we also define the signal *u*_*n*_.

*u*_*n*_ (deg): Visuomotor distortion. This is the negative of the visuomotor rotation

A4 $$ u_{n} = - r_{n} . $$

*e*_*n*_ (deg): Visual feedback error. The visuomotor distortion is not directly accessible. Therefore, the adaptation to this distortion is indirectly driven by the visual feedback error. It is defined as the difference between a visual reference orientation (${\widehat{h}}_{n}$) and the actual feedback (*h*_*n*_). Like the visuomotor distortion, positive values of *e*_*n*_ induce positive (clockwise) changes of the visually guided pointing direction. Consequently, *e*_*n*_ is defined as

A5 $$ e_{n} = \hat{h}_{n} - h_{n} . $$

With this definition, the visual reference orientation ${\widehat{h}}_{n}$ is implicitly defined as the visual feedback that, when consistently applied, leads finally to the baseline pointing direction (*g*_*n*_). Thereby, ${\widehat{h}}_{n}$ is not necessarily a deterministic variable but may be interpreted as a random variable (like the actual visual feedback *h*_*n*_). This seems natural because the reference orientation ${\widehat{h}}_{n}$ has been interpreted previously as the “expected visual feedback” (Synofzik et al. 2006, 2008) represented by neural activity. Therefore, we do not assume that ${\widehat{h}}_{n}={g}_{n}$ but only that the expectation of the random variable ${\widehat{h}}_{n}$ equals the motor goal *g*_*n*_. Another reason to assume that ${\widehat{h}}_{n}$ is a random variable is that previous studies suggested that error sources affecting the actual visual feedback, such as transient external perturbation sources (Wei and Körding 2009) or errors due to planned exploration (Collins and Wallman 2012) can be ignored and do not contribute to adaptation. To achieve this, the expected visual feedback must account for the ignored error components. Our model does this by assuming that the visual reference orientation ${\widehat{h}}_{n}$ is derived from the motor goal by adding a certain fraction (*q*) of the execution noise *v* (0*q*1).

*q*: Noise-compensation gain:

A6 $$ \hat{h}_{n} = g_{n} + q v_{n} . $$

The visual feedback error *e*_*n*_ drives the adaptation in the following way:

A7a $$ \underline {x}_{n + 1} = \user2{A }\underline {x}_{n} + \underline {b} e_{n} + \underline {w}_{n} $$

A7b $$ d_{n} = g_{n} + \underline {c}^{T} \underline {x}_{n} + v_{n} $$

where ***A*** is a diagonal 2 × 2 matrix with the retention rates $\underline{a}=\left[{a}_{s};{a}_{f}\right]$ applied to the slow and fast memory state, i.e., the components of the state vector ${\underline{x}}_{n}$. The vector $\underline{b}=\left[{b}_{s};{b}_{f}\right]$ is a 2-dimensional column vector containing the error sensitivities of the slow and the fast memory state, and the two-dimensional random vector ${\underline{w}}_{n}$ denotes the two noise components added to the memory states. This reflects the idea that the generation of a specific internal motor plan (in this case that of a visually guided movement) is adapted with respect to a memory of its previous state and that this memory is subject to noise (van Beers 2009). We assume here that ${\underline{w}}_{n}$ is normally distributed with zero mean and a diagonal matrix of covariance

A8 $$ \underline {w}_{n} \sim N\left( {\underline {0} ,{ }{\varvec{W}}} \right){ ; } {\varvec{W}} = \left[ {\begin{array}{*{20}c} {w_{ss}^{2} } & {w_{sf}^{2} } \\ {w_{sf}^{2} } & {w_{ff}^{2} } \\ \end{array} } \right]. $$

In the simplest version of the model, it is assumed that ***W*** is constant (independent of time) like ${\sigma }^{2}$, the variance of the execution noise. The scalar product ${\widehat{u}}_{n}={\underline{c}}^{T} {\underline{x}}_{n}$ on the right side of (Eq. A7b) denotes the projection of the memory states onto the motor output. The adaptive change ${\widehat{u}}_{n}={\underline{c}}^{T} {\underline{x}}_{n}$ of the adaptation dynamics can be interpreted as an estimator of the visuomotor distortion *u*_*n*_. This estimate is added to the motor goal to obtain the visually guided pointing direction ${vd}_{n}$, which is then contaminated by the execution noise ${v}_{n}$ to obtain the actual pointing direction ${d}_{n}$. The projection weight ${\underline{c}}^{T}$ is a row vector with both elements identical to one. This assumption is motivated by the problem that the relation between input (${e}_{n}$) output (${d}_{n}$) of this system is unaffected by linear transformations of the filter coefficients of the type $\stackrel{\sim }{{\varvec{A}}}={\varvec{T}} {\varvec{A}} {{\varvec{T}}}^{-1}, \stackrel{\sim }{\underline{b}}={\varvec{T}}\underline{b}, {\underline{\stackrel{\sim }{c}}}^{T}={\underline{c}}^{T}{{\varvec{T}}}^{-1}$. Thus, the assumption of $\underline{c}$ being fixed avoids ambiguities in model identification.

*y*_*n*_ (deg): relative pointing direction with respect to baseline. This angle is defined as the difference between the pointing direction *d*_*n*_ and the motor goal

A9 $$ y_{n} = d_{n} - g_{n.} $$

This definition is useful because it allows the adaptive process to be expressed independent of the direction of the motor goal as follows. For closed-loop trials, when the visual feedback signal *h*_*n*_ depends on the movement of the subject, Eqs. (A1–A7) can be used to express the feedback error in relation to the state vector:

A10 $$ e_{n} = u_{n} - \underline {c}^{T} \underline {x}_{n} + \left( {q - 1} \right)v_{n} . $$

Inserting Eqs. (A9) and (A10) in Eq. (A7a/b) leads then to the following time discrete filter

A11a $$ \underline {x}_{n + 1} = \left( {{\varvec{A}} - \underline{b} \ \underline{\text{c}}^{T} } \right) \underline {x}_{n} + \underline {b} { }\left( {u_{n} + \left( {q - 1} \right) v_{n} } \right) + \underline {w}_{n} $$

A11b $$ y_{n} = \underline {c}^{T} \underline {x}_{n} + v_{n} . $$

In error-clamp trials, the visual feedback does not depend on the subject’s action but is determined by the experimenter. The cursor moves at a fixed angle with respect to the target direction and consequently also at a fixed angle (${-\delta }_{n}$) with respect to the motor goal

A12 $$ h_{n} = g_{n} - \delta_{n} . $$

According to Eqs. (A5/A6/A12), the feedback error evaluates in error-clamp trials to

A13 $$ e_{n} = \delta_{n} + q v_{n} , $$

, and Eq. (A11a) must be replaced in error-clamp trials by

A14 $$ \underline {x}_{n + 1} = \user2{A }\underline {x}_{n} + \underline {b} { }\left( {\delta_{n} + q v_{n} } \right) + \underline {w}_{n} . $$

Following Albert and Shadmehr (2017), we also consider set breaks that may occur after a closed-loop trial or after an error-clamp trial with the index *n*. In those cases, the state updates for the rial *n* + 1 result from Eqs. (A11a) and (A13), by left-multiplying the right side of these equations by a matrix ***D***_*n*_ which is assumed to be a diagonal matrix of the form

A15 $$ {\varvec{D}}_{n} = \left[ {\begin{array}{*{20}c} {a_{s} } & 0 \\ 0 & {a_{f} } \\ \end{array} } \right]^{{p_{n} }} , $$

where *p*_*n*_ is larger than zero for set breaks after the trial *n* and equals zero otherwise. Equations (A11a/b, 13), and (A15) can be put together into a single time-variant discrete system of the form. For greater generality, we use here an output gain-vector ${\underline{c}}_{n}$ that is also time-dependent:

A16a $$ \underline {x}_{n + 1} = {\varvec{A}}_{n} \user2{ }\underline {x}_{n} + \underline {b}_{n} { }\left( {z_{n} + {\upgamma }_{n} v_{n} } \right) + {\varvec{D}}_{n} \user2{ }\underline {w}_{n} $$

A16b $$ y_{n} = \underline {c}_{n}^{T} \underline {x}_{n} + v_{n} , $$

where ${\varvec{A}}_{n}$ denotes the time-dependent system matrix

A16c $$ {\varvec{A}}_{n} = \left\{ {\begin{array}{*{20}l} {{\varvec{D}}_{n} \user2{ }\left[ {\begin{array}{*{20}c} {a_{s} - b_{s} ,} & { - b_{s} } \\ { - b_{f} ,} & {a_{f} - b_{f} } \\ \end{array} } \right]{ },} \hfill & {\text{for closed loop trials}} \hfill \\ {{\varvec{D}}_{n} \user2{ }\left[ {\begin{array}{*{20}c} {a_{s} ,} & 0 \\ {0 ,} & {a_{f} } \\ \end{array} } \right],} \hfill & {\text{for error - clamp trials}} \hfill \\ \end{array} } \right. $$

*b*_*n*_ denotes the time-dependent input gain

A16d $$ \underline {b}_{n} \user2{ = D}_{n} \user2{ }\underline {b} , $$

*z*_*n*_ denotes the deterministic input

A16e $$ z_{n} = \left\{ {\begin{array}{*{20}c} {{\text{u}}_{n} ,} & {{\text{for }}\;{\text{losed }}\;{\text{loop }}\;{\text{trials}}} \\ {\delta_{n} ,} & {{\text{for }}\;{\text{error - clamp }}\;{\text{trials}}} \\ \end{array} } \right., $$

and ${\gamma }_{n}$ denotes the transfer gain of the execution noise into the feedback error *e*_*n*_

A16f $$ \gamma_{n} = \left\{ {\user2{ }\begin{array}{*{20}l} {q - 1,} \hfill & {{\text{for}}\;{\text{ closed}}\;{\text{ loop }}\;{\text{trials}}} \hfill \\ {q,} \hfill & {{\text{for}}\;{\text{ error - clamp}}\;{\text{ trials}}} \hfill \\ \end{array} } \right. $$

Concerning the model predictions of the expected output ${\left\{\overline{y}\right\}}_{0}^{N-1}$, Eq. (A16a–f) is identical with the standard two-rate model mentioned above (Smith et al. 2006). The new aspect of the formulation given in Eq. (A16) is that the state update (Eq. A16a) is contaminated by both execution noise (${v}_{n}$) and state noise (${\underline{w}}_{n}$), whereas the system output (Eq. A16b) is only disturbed by execution noise. In addition, the noise-compensation gain (*q*) allows partial compensation of the adaptation input for execution noise to be taken into account.

In the literature (Smith et al. 2006), the filter coefficients (${a}_{s}, {a}_{f}, {b}_{s}, {b}_{f}$) are often estimated by computing the expected output (${\overline{y}}_{n}$) of the model by simulating Eq. (A16a/b) in the absence of noise (${v}_{n}=0$, ${\underline{w}}_{n}=0$) and by minimizing the sum of squared residuals

A17 $$ \mathop \sum \limits_{n = 0}^{N - 1} \left( {y_{n} - \overline{y}_{n} } \right)^{2} = min. $$

This method of least squares (LSQ) is optimal in the sense of minimum variance estimates only if the sequence of the residual${\left\{y-\overline{y}\right\}}_{0}^{N-1}$ is a stationary and white random process. In this case, minimizing the sum of squared residuals maximizes the likelihood of the observed output. However, for a system modeled as above, this would only be the case in the absence of the state noise (${\varvec{W}}=0$) and if the execution noise did not contribute to the feedback error (γ_*n*_ = 0). Therefore, LSQ provides estimates of the filter coefficients at a lower precision than a maximum-likelihood estimator based on the correct variance model.^2^ Beside the potential benefits of a correct variance model on the precision of the estimated filter coefficients, correctly estimating the variance components ***W*** and σ^2^ is an interesting question because it allows central and peripheral sources of execution noise to be distinguished (van Beers 2009). The development of a suitable variance model and of the corresponding maximum-likelihood estimator is therefore important for quantitative understanding of visuomotor adaptation. Previous studies (Tanaka et al. 2012; Albert and Shadmehr 2017) have already used maximum-likelihood methods based on the standard Kalman observer. The next section describes this approach and its advantages and drawbacks.

### A2: The standard Kalman observer

The standard Kalman approach for optimal state estimates of linear dynamic systems from noisy output observations (Kalman 1960) is made under the assumption of two distinct and independent noise sources: measurement noise and process noise. It assumes that the internal states of the system are iteratively updated by multiplication with the system matrix ***A*** and linear summation with a weighted input signal ${s}_{n}$. In addition, the state update is contaminated by the process noise ${\underline{w}}_{n}$:

A18a $$ \underline {x}_{n + 1} = \user2{A }\underline {x}_{n} + \underline {b} s_{n} + \underline {w}_{n} $$

The scalar output (${y}_{n}$) is the weighted sum of the process states (${\underline{x}}_{n}$), contaminated by the measurement noise ${v}_{n}$:

A18b $$ y_{n} = \underline {c}_{ n}^{T} \underline {x}_{n} + v_{n} $$

In addition, the standard Kalman observer assumes that both measurement noise and process noise are Gaussian with zero-mean and that both are independent of each other:

A19 $$ \left[ {\begin{array}{*{20}c} {v_{n} } \\ {\underline {w}_{n} } \\ \end{array} } \right]\sim N\left( {\left[ {\begin{array}{*{20}c} 0 \\ {\underline {0} } \\ \end{array} } \right],\left[ {\begin{array}{*{20}c} {\sigma^{2} } & {\underline {0}^{T} } \\ {\underline {0} } & {\varvec{W}} \\ \end{array} } \right]} \right) $$

For this system, the state vector ${\underline{x}}_{n}$ is also normally distributed because it results from linear operations on the deterministic signal ${s}_{n}$ and the normally distributed process noise ${w}_{n}$.

A20 $$ \underline {x}_{n} \sim N\left( {\overline{{\underline {x} }}_{n} ,{ }{{\varvec{\Sigma}}}_{n} } \right), {{\varvec{\Sigma}}}_{n} = \left[ {\begin{array}{*{20}c} {\sigma_{s n}^{2} } & {\sigma_{sf n}^{2} } \\ {\sigma_{sf n}^{2} } & {\sigma_{f n}^{2} } \\ \end{array} } \right] $$

The Kalman observer provides an iterative update of the mean and the covariance matrix of the states. Thus, the Kalman observer provides with ${\overline{\underline{x}}}_{n+1}$ and ${{\varvec{\Sigma}}}_{n+1}$ the time course of the prior distribution of the state vector ${\underline{x}}_{n+1}$ conditioned on all previous observations ${\left\{y\right\}}_{0}^{n}$. This iterative update is performed in two steps per trial. First, the prior estimates are updated by the information provided by the measurement of ${y}_{n}$, yielding the so-called posterior estimates of the means (${\overline{\underline{x}}}_{n}^{p}$) and covariance matrix (${{\varvec{\Sigma}}}_{n}^{p}$) of the state vector:

A21a $$ \underline {k}_{n} = {{\varvec{\Sigma}}}_{n} \underline {c}_{ n} /\left( {\underline {c}_{ n}^{T} {{\varvec{\Sigma}}}_{n} \underline {c}_{ n} { } + { }\sigma^{2} } \right) $$

A21b $$ \underline{{\overline{x}}}_{n}^{p} = \overline{{\underline {x} }}_{n} + \underline {k}_{n} \left( {y_{n} - \underline {c}_{ n}^{T} \cdot \overline{{\underline {x} }}_{n} } \right) $$

A21c $$ {{\varvec{\Sigma}}}_{n}^{p} = {{\varvec{\Sigma}}}_{n} - \underline {k}_{n} \user2{ }\underline {c}_{ n}^{T} {{\varvec{\Sigma}}}_{n} $$

The elements of the vector *k* denote the so-called Kalman gains, i.e., the sensitivity of the posterior mean states with respect to the difference between the actual and expected observation. In the second step of the iteration, the posterior estimates are updated to the prior estimates of the next time step *n* + 1 by considering the system dynamics and the process noise:

A22a $$ \overline{{\underline {x} }}_{n + 1} = \user2{A }\overline{{\underline {x} }}_{n}^{p} + \underline {b} s_{n} $$

A22b $$ {{\varvec{\Sigma}}}_{n + 1} = \user2{A }{{\varvec{\Sigma}}}_{n}^{p} {\varvec{A}}^{{\varvec{T}}} + {\varvec{W}} $$

The iteration Eqs. (A21/A22) start with the distribution parameters of the initial states *x*_*0*_:

A23 $$ \underline {x}_{0} \sim N\left( {\overline{{\underline {x} }}_{0} ,{ }{{\varvec{\Sigma}}}_{0} } \right) $$

For a given set of system parameters $\underline{\vartheta }=\left[{\varvec{A}}, \underline{b}, {\sigma }^{2}, {\varvec{W}}, {{\varvec{\Sigma}}}_{0}, {\underline{\overline{x}}}_{0}\right]$, the prior distributions of ${\underline{x}}_{n}$, provided by the Kalman observer, can be used to compute the negative log-likelihood (N*LL*) of the observation (${\left\{y\right\}}_{0}^{N-1}$) conditioned on these parameters:

A24 $$ NLL = - {\text{log}}\left( {L\left( {\left. {\left\{ y \right\}_{0}^{N - 1} } \right|, \underline {\vartheta } } \right)} \right) = \frac{1}{2} \mathop \sum \limits_{n = 0}^{N - 1} \frac{{\left( {y_{n} - \underline {c}_{ n}^{T} \overline{{\underline {x} }}_{n} } \right)^{2} }}{{\underline {c}_{ n}^{T} {{\varvec{\Sigma}}}_{n} \underline {c}_{ n} { } + { }\sigma^{2} }} + \frac{1}{2} \mathop \sum \limits_{n = 0}^{N - 1} {\text{log}}\left( {\underline {c}_{ n}^{T} {{\varvec{\Sigma}}}_{n} \underline {c}_{ n} { } + { }\sigma^{2} } \right) + \frac{N}{2} {\text{log}}\left( {2{ }\pi } \right) $$

Minimizing the negative log-likelihood (Eq. A24) can then be used not only to fit the system’s filter coefficients ***A***, *b*, and the expectation of the initial state vector $$\left({\overline{\underline{x}}}_{0}=\left[\begin{array}{c}{\overline{x}}_{s0}\\ {\overline{x}}_{f0}\end{array}\right]\right)$$, but also the variance parameters (${\sigma }_{v}^{2}$, ***W***, ${\boldsymbol{\Sigma }}_{0}$).

Previous studies (Albert and Shadmehr 2017; Tanaka et al. 2012; Zarahn et al. 2008) minimized the negative log-likelihood (Eq. A24) of data acquired in the context of an adaptation experiment to visuomotor rotation (the same type of adaptation investigated by the current study). They used the same two-rate adaptation model (Smith et al. 2006; Criscimagna-Hemminger and Shadmehr 2008) described above and minimized the likelihood of the observed pointing directions with respect to the error sensitivities ($$\underline{b}=\left[\begin{array}{c}{b}_{s}\\ {b}_{f}\end{array}\right]$$) and the two retention factors ($${\varvec{A}}=\left[\begin{array}{cc}{a}_{s}& 0\\ 0& {a}_{f}\end{array}\right]$$). The visual feedback error (${e}_{n}$) was identified with the system’s input signal ${s}_{n}$ (Eq. A18a), and the relative pointing direction with respect to the baseline represented the system’s output sequence ${y}_{n}$ (Eq. A18b).

The above-mentioned studies differed in that Zarahn et al. (2008), as well as Tanaka et al. (2012) minimized the negative log-likelihood directly, whereas Albert and Shadmehr (2017) used an expectation–maximization (EM) algorithm (Dempster et al. 1977). However, this difference concerns mainly the convergence properties of the applied algorithms because the EM-algorithm converges at the same solution as the maximum-likelihood approach. In the following, we describe the various aspects of the variance structure defined by Eq. (A16) that are simplified by this approach.

First, by identifying the feedback error ${e}_{n}$ with the input signal ${s}_{n}$ of the standard Kalman approach ignores the fact that the input to the state update equation (${z}_{n}+{\upgamma }_{n}\bullet {v}_{n}$, Eq. A16a) is deterministic only for error-clamp trials with zero noise-compensation gain (*q* = 0). In all other cases, the stochastic nature of the system input ${z}_{n}+{\upgamma }_{n}\bullet {v}_{n}$ will cause the distribution of the output ${\left\{y\right\}}_{0}^{N-1}$ to differ from that predicted by the simplified model. Second, in the simplified model (Eqs. A18), the system matrix ***A*** is the same for error-clamp and closed-loop trials, whereas in the more general formulation of the adaptation model (Eq. A16), the system matrix ***A***_*n*_ depends on the trial type. We therefore expect that the asymptotic covariance matrix (${{\varvec{\Sigma}}}_{\infty }$) of the system states differs between trial types, whereas the simplified model does not predict such a difference. Third, the variance of the noise (*w*_*n*_, Eq. A18a) entering the simplified state update does not depend on the trial type. In contrast, the sum of the noise components entering the state update of the more general model (${\underline{w}}_{n}+\underline{b}\bullet {\gamma }_{n}\bullet {v}_{n}$, Eq. A16a) depends on the trial type because the transfer gain of the execution noise into the feedback error (${\gamma }_{n}$) depends on the trial type. Equation (A16f) shows that a dependence of the state noise on the trial type exists independent of the assumptions about the noise compensation gain (0 ≤ *q* ≤ 1). This is another reason why the general but not the simplified model predicts that ${\left.{{\varvec{\Sigma}}}_{n}\right|}_{n\to \boldsymbol{\infty }}$ depends on the trial type. In conclusion, previous applications of the Kalman observer for computing the likelihood of observations in visuomotor adaptation were not consistent with the variance/covariance structure defined by Eq. (A16) because these approaches did not consider that in visuomotor adaptation the peripheral execution noise reenters the system’s input via the error signal.

In the current study, we want to develop a maximum-likelihood estimation method for the variance structure defined by the model as defined by Eq. (A16). Therefore, we must clarify how the above Kalman observer can be extended to be applied to the closed-loop model. The dependence of the system matrix and of the state noise on time can easily be included in the standard Kalman approach by supplying ***A*** or the ***W*** in Eq. (A22a/b) with the index *n*. In contrast, the transfer of execution noise to the state update constitutes a problem for the standard Kalman approach because it causes the state noise (${\underline{w}}_{n}+\underline{b}\bullet {\gamma }_{n}\bullet {v}_{n}$, Eq. A16a) and the measurement noise (${v}_{n}$, Eq. A16b) to depend statistically on each other. This violates an important precondition (Eq. A19) of the standard Kalman observer and therefore prohibits its direct application for computing the likelihood of an observation ${\left\{y\right\}}_{0}^{N-1}$ of the adaptation system (Eq. A16).

These considerations underline the fact that previous approaches to maximum-likelihood estimators in visuomotor adaptation mark important progress in identifying the two-rate learning model compared to the simpler least-square approach. However, the use of the standard Kalman observer implied a simplification of the variance structure of the adaptation system that is not fully compatible with the system defined by Eq. (A16). To compute the exact likelihood of ${\left\{y\right\}}_{0}^{N-1}$ for this system, we developed two different methods. First, we will present an algorithm to compute the mean $\underline{\overline{y}}$ (dimension N × 1) of the observation vector $\underline{y} :={\left\{y\right\}}_{0}^{N-1}$ and its covariance matrix ***Y*** (dimension *N* x *N*). The inverse ${{\varvec{Y}}}^{-1}$ is then needed to compute the log-likelihood of the multivariate normal distribution of the observation$\underline{y}$. The second method, which is numerically much more efficient, will extend the standard Kalman observer to the case when the measurement noise (or a fraction of it) is fed back into the system’s input as in the model defined by Eq. (A16).

### A3: The variance/covariance structure of the observation

Testing the variance/covariance structure of the pointing directions predicted by the model (Eq. A16) requires a comparison of the expected covariance matrix (${\varvec{Y}}={[{Y}_{i, j}]}_{i, j=0}^{N-1}$) with the empiric data ($\underline{y}$). Ideally, to estimate the empiric covariance matrix, it would be desirable to observe repeated adaptation time courses in the same subject under identical conditions, i.e., with identical initial conditions of the internal visuomotor memory and with the same underlying adaptation dynamics. Such empirical data are difficult to obtain, first, because the memory states are affected by previous experiences and, second, because it was also suggested that the adaptation dynamics depend on previous experience (Herzfeld et al. 2014; Huang and Shadmehr 2009). Like most previous studies, we did not acquire many repeated adaptations in a single subject but estimated the characteristics of the within-subject noise by evaluating the variance and the autocovariance of the residual error $\left({r}_{n}={y}_{n}-{\overline{y}}_{n}\right)$ within a window of length *N* = 2*w* + 1 centered around the trial *n*. These empiric measures were compared with the corresponding model predictions that were obtained as follows: For the given trial window, the expected autocovariance at lag $\Delta$ can be derived from the expected covariance matrix ***Y***

A25 $$ \begin{aligned} \overline{acov} \_r_{n} \left( {\Delta } \right) & = E\left\{ {\frac{1}{{N - {\Delta }}}\mathop \sum \limits_{k = n - w}^{{n + w - {\Delta }}} \left( {r_{k} - \frac{1}{{N - {\Delta }}}\mathop \sum \limits_{i = n - w}^{{n + w - {\Delta }}} r_{i} } \right) } \right.\left. { \cdot \left( {r_{{k + {\Delta }}} - \frac{1}{{N - {\Delta }}}\mathop \sum \limits_{j = n - w}^{{n + w - {\Delta }}} r_{{i + {\Delta }}} } \right)} \right\} \\ & \quad = \frac{1}{{N - {\Delta }}}\left( {\mathop \sum \limits_{k = n - w}^{{n + w - {\Delta }}} Y_{{k, k + {\Delta }}} } \right) - \frac{1}{{\left( {N - {\Delta }} \right)^{2} }}\left( {\mathop \sum \limits_{i, j = n - w}^{{n + w - {\Delta }}} Y_{{i, j + {\Delta }}} } \right) \\ \end{aligned} $$

where ${Y}_{i, j}$ denotes the element of ***Y*** in row *i* and column *j*. As a special case, we obtain also the expected variance of the residual (${\overline{var}\_r}_{n}={\overline{acov}\_r}_{n}(\Delta =0)$) in this window. We evaluated the time course of the observed residual variance and autocovariance in a running window with the length of 20 trials and compared it with the corresponding model predictions obtained by Eq. (A25).

To test this model prediction of the expected autocovariance, we need to evaluate the covariance matrix ***Y***. This was achieved by the following algorithm. Within a first loop, the expectation ${\left\{\overline{\underline{x}}\right\}}_{0}^{N-1}$ of the states, their covariance matrices ${\left\{{{\varvec{\Sigma}}}_{n}\right\}}_{0}^{N-1}$, the expected observation $\underline{\overline{y}}={\left\{\overline{y}\right\}}_{0}^{N-1}$, and the diagonal elements ${\left\{{Y}_{i,i}\right\}}_{i=0}^{N-1}$ were computed. The iteration started with

A26 $$ \overline{y}_{0} = \underline {c}_{ 0}^{T} \underline{{\overline{x}}}_{0} , Y_{0, 0} = \underline {c}_{ 0}^{T} {{\varvec{\Sigma}}}_{0} \underline {c} _{0} + \sigma^{2} $$

and proceeded with

A27a $$ \underline{{\overline{x}}}_{n + 1} = A_{n} \overline{{\underline {x} }}_{n} + \underline {b}_{n} z_{n} , \overline{y}_{n + 1} = \underline {c}_{ n + 1}^{T} \underline{{\overline{x}}}_{n + 1} $$

A27b $$ {{\varvec{\Sigma}}}_{n + 1} = {\varvec{A}}_{n} { }{{\varvec{\Sigma}}}_{n} {\varvec{A}}_{n}^{T} + {\varvec{D}}_{n} {\varvec{W}} {\varvec{D}}_{n}^{T} + \gamma_{n}^{2} \sigma^{2} \underline {b}_{n} \underline {b}_{n}^{T} $$

A27c $$ Y_{n + 1,n + 1} = \underline {c}_{ n + 1}^{T} {{\varvec{\Sigma}}}_{n + 1} \underline {c}_{ n + 1} + \sigma^{2} . $$

Then, the non-diagonal elements of ***Y*** were computed as follows

A28a $$ {\varvec{cov}}\left( {\underline {x}_{n + k} , \underline {x}_{n} } \right) = {\varvec{A}}_{n + k - 1} {\varvec{A}}_{n + k - 2} {\varvec{A}}_{n + k - 3} \ldots {\varvec{A}}_{n} {{\varvec{\Sigma}}}_{n} ,\;\;\;{\text{for}}\;{1} \le k \le n - 1, $$

A28b $$ Y_{n + 1,n} = \underline {c}_{ n + 1}^{T} {\varvec{cov}}\left( {\underline {x}_{n + 1} , \underline {x}_{n} } \right) \underline {c}_{n} + \underline {c}_{ n + 1}^{T} \underline {b}_{n} \gamma_{n} \sigma^{2} , $$

A28c $$ \begin{aligned} Y_{n + k,n} & = \underline {c}_{ n + k}^{T} {\varvec{cov}}\left( {\underline {x}_{n + k} , \underline {x}_{n} } \right) \underline {c}_{n} \\ & \quad + \underline {c}_{ n + k}^{T} {\varvec{A}}_{n + k - 1} {\varvec{A}}_{n + k - 2} \ldots {\varvec{A}}_{n + 1} \underline {b}_{n} \gamma_{n} \sigma^{2} \;\;\;{\text{for 2}} \le k \le N - n - 1. \\ \end{aligned} $$

The elements of ***Y*** above the diagonal were obtained from the symmetry feature of covariance matrices (${Y}_{j, j}={Y}_{j, i}$).

The asymptotic covariance matrix (${{\varvec{\Sigma}}}_{\infty }$) of the states can be computed from Eq. (A27b) by

A29 $$ {{\varvec{\Sigma}}}_{\infty } = {\varvec{V}}\left( {\user2{ }\left( {{\varvec{V}}^{ - 1} \user2{ }\left( { {\varvec{W}} + \gamma_{\infty }^{2} \sigma^{2} \underline {b}_{\infty } \underline {b}_{\infty }^{T} } \right) {\varvec{V}}^{ - 1^{T}} } \right) \cdot /{\varvec{E}} - \underline {\lambda } \underline {\lambda }^{T} } \right){\varvec{V}}^{T} , $$

where ***V*** denotes the matrix of eigenvectors of${{\varvec{A}}}_{\infty }$, $\underline{\lambda }$ denotes the vector of eigenvalues of ${{\varvec{A}}}_{\infty }$ and./ denotes elementwise division. We exclude here the occurrence of set-break trials in the asymptotic system by assuming that${{\varvec{D}}}_{\infty }={\varvec{E}}$. The resulting ${{\varvec{\Sigma}}}_{\infty }$ can be used to compute the asymptotic variance of the output ${y}_{\infty }$ by inserting ${{\varvec{\Sigma}}}_{\infty }$ in Eq. (A27c). The asymptotic autocovariance function *ACF*(*Δ*)=${Y}_{\infty +\Delta , \infty }$ is computed by inserting ${{\varvec{\Sigma}}}_{\infty }$ in Eq. (A28a–c). For the special case of a one-rate adaptation model during closed-loop condition without noise-compensation (*γ* =  − 1), and with perfect retention (*a* = 1), van Beers (2009) provided the formulas for the asymptotic variance and the *ACF*(1). We verified that our formulas are, for this special case, identical with those of van Beers (2009). Thus, the method presented above is a generalization of van Beers (2009) formula to multi-rate adaptation models and allows $\underline{\overline{y}}$ and ***Y*** to be computed for a large variety of experimental designs including non-stationary ones composed of arbitrary sequences of closed-loop trials, error-clamp trials, and set-break trials.

Once the mean $\underline{\overline{y}}$ and the covariance matrix ***Y*** of the observation are known for a given set of model parameters, the negative log-likelihood (NLL) of an observation $\underline{\overline{y}}$ can be computed by

A30 $$ \begin{aligned} NLL & = - log\left( {L\left( {\left. {\left\{ y \right\}_{0}^{N - 1} } \right|, \underline {\vartheta } } \right)} \right) = \frac{1}{2} \left( {\underline {y} - \overline{{\underline {y} }} } \right)^{T} \cdot {\varvec{Y}}^{ - 1} \cdot \left( {\underline {y} - \overline{{\underline {y} }} } \right) \\ & \quad + \frac{1}{2}{\text{ log}}\left( {\det \left( {\varvec{Y}} \right)} \right) + \frac{N}{2} \cdot \log \left( {2 \cdot \pi } \right). \\ \end{aligned} $$

If any of the observations ${y}_{n}$ are missing or invalid, the log-likelihood can be computed using the same formula Eq. (A30) after deleting the missing elements from the vectors $\underline{y}$, $\overline{\underline{y}}$, after deleting the corresponding rows and columns of ***Y***, and after reducing *N* by the number of missing ${y}_{n}$. Equation (A30) allows the negative log-likelihood of the output sequence $\underline{\overline{y}}$ for the system Eq. (A16) to be computed even though this closed-loop system cannot be optimally observed by the standard Kalman approach.

Unfortunately, using Eq. (A30) to compute the negative log-likelihood is rather inefficient because the required inversion of the *N* × *N* matrix ***Y*** is a time-consuming procedure. Therefore, in the following section, we extended the standard Kalman observer to estimate the likelihood of the model Eq. (A16).

### A4: Kalman observer for recursive adaptive systems

Adaptive systems typically learn from errors resulting from their own actions. Therefore, peripheral noise, such as execution noise, usually feeds back into the process noise as modeled in Eq. (A16a–f). This noise feedback violates the assumption of the standard Kalman observer that the noise components contaminating the state-update and the model output are independent as in Eq. (A18a/b). To generalize the Kalman observer to the closed-loop situation of Eq. (A16a–f), we derive here its update equations by the method used by Nakata and Tonetti (2010). The essential idea of this method is to compute the prior distribution of $\left(\left.{\underline{x}}_{n+1}\right| {\left\{y\right\}}_{0}^{n}\right),$ conditioned on all previous observations of ${\left\{y\right\}}_{0}^{n},$ from the joint distribution of

A31 $$ \left( {\left. {\left[ {\begin{array}{*{20}c} { \underline {x}_{n + 1} } \\ { y_{n} } \\ \end{array} } \right] } \right|\left\{ y \right\}_{0}^{n - 1} } \right) \sim N\left( {\underline {m}_{n + 1, n} , {\varvec{S}}_{n + 1, n} } \right) $$

, which is normally distributed with the mean

A32 $$ \underline {m}_{n + 1, n} = \left[ {\begin{array}{*{20}c} {{\varvec{A}}_{n} \underline{{\overline{x}}}_{n} + \underline {b}_{n} z_{n} } \\ {\underline {c}_{ n}^{T} \underline{{\overline{x}}}_{n} } \\ \end{array} } \right] $$

and the covariance matrix

A33 $$ \user2{\underline {S} }_{n + 1, n } = \left[ {\begin{array}{*{20}l} {{\varvec{A}}_{n} { }{{\varvec{\Sigma}}}_{n} {\varvec{A}}_{n}^{T} + {\varvec{D}}_{n} {\varvec{W}} {\varvec{D}}_{n}^{T} + \gamma_{n}^{2} \sigma^{2} \underline {b}_{n} \underline {b}_{n}^{T} ,} \hfill & {{\varvec{A}}_{n} { }{{\varvec{\Sigma}}}_{n} \underline {c}_{n} + \gamma_{n} \underline {b}_{n} \sigma^{2} } \hfill \\ {\underline {c}_{n}^{T} {{\varvec{\Sigma}}}_{n} {\varvec{A}}_{n}^{T} + \gamma_{n} \underline {b}_{n}^{T} \sigma^{2} ,} \hfill & {\underline {c}_{n}^{T} {{\varvec{\Sigma}}}_{n} \underline {c}_{n} + \sigma^{2} } \hfill \\ \end{array} } \right]. $$

Note that both Eqs. (A32) and (A33) are consistent with Eq. (A27). The conditional distribution of $\left(\left.{\underline{x}}_{n+1}\right| {\left\{y\right\}}_{0}^{n}\right)$ is now identified from Eqs. (A32) and (A33) by using the following lemma about conditional normal distributions:

If $$\left[\begin{array}{c}{\underline{x}}_{1}\\ {\underline{x}}_{2}\end{array}\right]$$ is normally distributed

A34a $$ \left[ {\begin{array}{*{20}c} {\underline {x}_{1} } \\ {\underline {x}_{2} } \\ \end{array} } \right]\sim N\left( {\left[ {\begin{array}{*{20}c} {\underline {\mu }_{1} } \\ {\underline {\mu }_{2} } \\ \end{array} } \right] , \left[ {\begin{array}{*{20}c} {{{\varvec{\Sigma}}}_{11} } & {{{\varvec{\Sigma}}}_{12} } \\ {{{\varvec{\Sigma}}}_{21} } & {{{\varvec{\Sigma}}}_{22} } \\ \end{array} } \right]} \right), $$

then the conditional distribution of $\left.{\underline{x}}_{1}\right| {\underline{x}}_{2}$ is

A34b $$ \left. {\underline {x}_{1} } \right| \underline {x}_{2} \sim N\left( {\underline {\mu }_{1} + {{\varvec{\Sigma}}}_{12} {{\varvec{\Sigma}}}_{22}^{ - 1} \left( {\underline {x}_{2} - \underline {\mu }_{2} } \right) , {{\varvec{\Sigma}}}_{11} - {{\varvec{\Sigma}}}_{12} {{\varvec{\Sigma}}}_{22}^{ - 1} {{\varvec{\Sigma}}}_{21} } \right). $$

Applying this lemma to the joint distribution of Eqs. (A31), (A32), and (A33) yields

A35 $$ \left( {\left. {\underline {x}_{n + 1} } \right| \left\{ y \right\}_{0}^{n} } \right) \sim N\left( {\underline{{\overline{x}}}_{n + 1} , {{\varvec{\Sigma}}}_{n + 1} } \right). $$

with

A36 $$ \begin{aligned} \overline{{\underline {x} }}_{n + 1} & = \user2{ A}_{n} \underline{{\overline{x}}}_{n} + \underline {b}_{n} z_{n} \\ & \quad + \left( {{\varvec{A}}_{n} { }{{\varvec{\Sigma}}}_{n} \underline {c}_{n} + \gamma_{n} \underline {b}_{n} \sigma^{2} } \right) \left( {\underline {c}_{n}^{T} {{\varvec{\Sigma}}}_{n} \underline {c}_{n} + \sigma^{2} } \right)^{ - 1} \left( {y_{n} - \underline {c}_{n}^{T} \underline{{\overline{x}}}_{n} } \right), \\ \end{aligned} $$

and

A37 $$ \begin{aligned} {{\varvec{\Sigma}}}_{n + 1} & = \user2{ A}_{n} { }{{\varvec{\Sigma}}}_{n} {\varvec{A}}_{n}^{T} + {\varvec{D}}_{n} {\varvec{W}} {\varvec{D}}_{n}^{T} + \gamma_{n}^{2} \sigma^{2} \underline {b}_{n} \underline {b}_{n}^{T} \\ & \quad - \left( {{\varvec{A}}_{n} { }{{\varvec{\Sigma}}}_{n} \underline {c}_{n} + \gamma_{n} \underline {b}_{n} \sigma^{2} } \right) \left( {\underline {c}_{n}^{T} {{\varvec{\Sigma}}}_{n} \underline {c}_{n}^{T} + \sigma^{2} } \right)^{ - 1} \left( {\underline {c}_{n}^{T} {{\varvec{\Sigma}}}_{n} {\varvec{A}}_{n}^{T} + \gamma_{n} \underline {b}_{n}^{T} \sigma^{2} } \right). \\ \end{aligned} $$

These two equations define the iterative update of the generalized Kalman observer. Starting with the initial condition Eq. (A23), we used this generalized Kalman observer to compute the means ${\left\{\underline{\overline{x}}\right\}}_{0}^{n-1}$ and the covariance matrices ${\left\{{\varvec{\Sigma}}\right\}}_{0}^{n-1}$ of the prior distributions for all trials and inserted them into Eq. (A24) to compute the negative log-likelihood of the observation ${\left\{y\right\}}_{0}^{N-1}$. It is easy to verify that for the special case ${\gamma }_{n}=0$, ${{\varvec{D}}}_{n}={\varvec{E}}$ the generalized Kalman update (Eqs. A36/A37) is identical to that of the standard Kalman observer (Eqs. A21/A22).

In case one or more subsequent measurements ${\left\{y\right\}}_{n+1}^{n+k}$ are missing (*k* > 0), the generalized Kalman observer must update the parameters of the prior distribution of $\left(\left.{\underline{x}}_{n}\right| {\left\{y\right\}}_{0}^{n-1}\right)$ to those of $\left(\left.{\underline{x}}_{n+1+k}\right| {\left\{y\right\}}_{0}^{n}\right)$. This is achieved by applying the lemma Eq. (A34) on the joint distribution of

A38 $$ \left( {\left. {\left[ {\begin{array}{*{20}c} { \underline {x}_{n + k + 1} } \\ { y_{n} } \\ \end{array} } \right] } \right|\left\{ y \right\}_{0}^{n - 1} } \right) \sim N\left( {\underline {m}_{n + k + 1, n} , {\varvec{S}}_{n + k + 1, n} } \right). $$

Mean and covariance matrix of this distribution are defined recursively starting with by $${\underline{m}}_{n+1, n}=\left[\begin{array}{c}{\underline{\mu }}_{xk}\\ {\mu }_{yk}\end{array}\right] , {{\varvec{S}}}_{n+1, n}=\left[\begin{array}{cc}{S}_{xxk} ,& {\underline{s}}_{ xyk}\\ {\underline{s}}_{ xyk}^{T} ,& {s}_{yyk}\end{array}\right]$$ identical to Eqs. (A32/A33) for k = 0 and proceed with

A39 $$ \underline {m}_{n + k + 1, n} = \left[ {\begin{array}{*{20}c} {{\varvec{A}}_{n + k} \underline {\mu }_{xk} + \underline {b}_{n + k} z_{n + k} } \\ {\mu_{y0} } \\ \end{array} } \right] $$

and

A40 $$ {\varvec{S}}_{n + k + 1, n} = \left[ {\begin{array}{*{20}l} {{\varvec{A}}_{n + k} S_{xxk} {\varvec{A}}_{n + k}^{T} + \user2{ D}_{n + k} {\varvec{W}} {\varvec{D}}_{n + k}^{T} + \gamma_{n + k}^{2} \sigma^{2} \underline {b}_{n + k} \underline {b}_{n + k}^{T} ,} \hfill & {{\varvec{A}}_{n + k} \underline {s}_{ xyk} } \hfill \\ { \underline {s}_{ xy0}^{T} {\varvec{A}}_{n + k}^{T} ,} \hfill & {s_{yyk} } \hfill \\ \end{array} } \right]. $$

Finally, application of lemma Eq. (A34) leads to the following generalized Kalman algorithm for computing the log-likelihood of the available observations ${\left\{y\right\}}_{0}^{n-1}$:

1. Set *n* = 0 and initialize ${\overline{\underline{x}}}_{n}$ and ${{\varvec{\Sigma}}}_{n}$ with the predefined values ${\overline{\underline{x}}}_{0}$ and ${{\varvec{\Sigma}}}_{0}$.
2. If ${y}_{n}$ is not missing, then compute the posterior estimates ${\underline{\overline{x}}}_{n}^{p}$ and ${{\varvec{\Sigma}}}_{n}^{p}$:
  A41a $$ \underline {k}_{n} = {{\varvec{\Sigma}}}_{n} \underline {c}_{ n} /\left( {\underline {c}_{ n}^{T} {{\varvec{\Sigma}}}_{n} \underline {c}_{ n} { } + { }\sigma^{2} } \right) $$
  A41b $$ \underline{{\overline{x}}}_{n}^{p} = \overline{{\underline {x} }}_{n} + \underline {k}_{n} \left( {y_{n} - \underline {c}_{ n}^{T} \cdot \overline{{\underline {x} }}_{n} } \right) $$
  A41c $$ {{\varvec{\Sigma}}}_{n}^{p} = {{\varvec{\Sigma}}}_{n} - \underline {k}_{n} \user2{ }\underline {c}_{ n}^{T} {{\varvec{\Sigma}}}_{n} $$
  and initialize the following auxiliary variables
  A42a $$ \underline {x}^{a} = \overline{{\underline {x} }}_{n}^{p} $$
  A42b $$ {{\varvec{\Sigma}}}^{a} = {{\varvec{\Sigma}}}_{n}^{p} $$
  A42c $$ \underline {k}^{a} = \gamma_{n} \underline {b}_{n} \sigma^{2} /\left( {\underline {c}_{ n}^{T} {{\varvec{\Sigma}}}_{n} \underline {c}_{ n} { } + { }\sigma^{2} } \right) $$
  A42d $$ \underline {\psi } = \underline {k}^{a} \left( {y_{n} - \underline {c}_{ n}^{T} \cdot \overline{{\underline {x} }}_{n} } \right) $$
  A42e $$ {{\varvec{\Gamma}}}_{1} = \underline {k}^{a} \underline {c}_{ n}^{T} {{\varvec{\Sigma}}}_{n} \user2{ A}_{n}^{T} $$
  A42f $$ {{\varvec{\Gamma}}}_{2} = \underline {k}^{a} \gamma_{n} \underline {b}_{n}^{T} \sigma^{2} . $$
3. Otherwise, if the measurement ${y}_{n}$ is missing, then update the auxiliary variables $\underline{\psi }$, ${{\varvec{\Gamma}}}_{1}$, and ${{\varvec{\Gamma}}}_{2}$:
  A43a $$ \underline {\psi } = {\varvec{A}}_{n} \underline {\psi } . $$
  A43b $$ {{\varvec{\Gamma}}}_{1} = {\varvec{A}}_{n} {{\varvec{\Gamma}}}_{1} \user2{ A}_{n}^{T} $$
  A43c $$ {{\varvec{\Gamma}}}_{2} = {\varvec{A}}_{n} {{\varvec{\Gamma}}}_{2} \user2{ A}_{n}^{T} . $$
4. Independent of whether ${y}_{n}$ is missing or not, update the auxiliary variables ${\underline{x}}^{a}$ and ${{\varvec{\Sigma}}}^{a}$*:*
  A44a $$ \underline {x}^{a} = {\varvec{A}}_{n} \underline {x}^{a} + \underline {b}_{n} z_{n} $$
  A44b $$ {{\varvec{\Sigma}}}^{a} = {\varvec{A}}_{n} {{\varvec{\Sigma}}}^{a} {\varvec{A}}_{n}^{T} + {\varvec{D}}_{n} {\varvec{W}} {\varvec{D}}_{n}^{T} + \gamma_{n}^{2} \sigma^{2} \underline {b}_{n} \underline {b}_{n}^{T} . $$
5. Compute the prior estimates ${\overline{\underline{x}}}_{n+1}$ and ${{\varvec{\Sigma}}}_{n+1}$:
  A45a $$ \overline{{\underline {x} }}_{n + 1} = \underline {x}^{a} + \underline {\psi } $$
  A45b $$ {{\varvec{\Sigma}}}_{n + 1} = {{\varvec{\Sigma}}}^{a} - {{\varvec{\Gamma}}}_{1} - {{\varvec{\Gamma}}}_{1}^{T} - {{\varvec{\Gamma}}}_{2} $$
6. Set *n* = *n* + 1 and go to 2) until *n* ≥ *N*.
7. Use the obtained ${\left\{\overline{\underline{x}}\right\}}_{0}^{N-1}$ and ${\left\{{\varvec{\Sigma}}\right\}}_{0}^{N-1}$ to evaluate the negative log-likelihood of the available observations ${\left\{y\right\}}_{0}^{n-1}$ by computing the sum of Eq. (A24) across all *n* for which ${y}_{n}$ is not missing.

This algorithm assumes that ${y}_{0}$ is not missing. Otherwise, the auxiliary variables needed in step 3 would be non-initialized. However, this assumption does not involve any loss of generality since the summation in step 7 starts with the *n* corresponding to the first non-missing ${y}_{n}$. Therefore, if the index of first valid measurement *y*_*nv*_ is positive (*nv* > 0), then replace all indices *n* by *n*-*nv* before starting the algorithm. Again, for ${\gamma }_{n}=0$ and ${{\varvec{D}}}_{n}={\varvec{E}}$, this algorithm based on the generalized Kalman observer is identical to that based on the standard Kalman observer (Eqs. A21/A22/A24).

We tested numerically that the negative log-likelihood $-log\left(L\left(\left.{\left\{y\right\}}_{0}^{N-1}\right|, \underline{{\vartheta }}\right)\right)$ provided by the above algorithm is the same as and is numerically more efficient than that provided by the method of the previous section (Eq. A30). For an experiment with 520 trials (*N*), the computing time of the Kalman-based method was about 30 times smaller.

### A5: Optimal observation of a recursive adaptive system with signal-dependent execution noise and planning noise

We considered two extensions of the model by replacing the white constant noise sources for planning noise and execution noise by time-variant noise sources consisting of a sum of constant and multiplicative noise.

The first type of model extension (M3) considered a time variant version of the execution noise ${v}_{n}$ consisting of a sum of constant and multiplicative noise where the multiplicative component was assumed to be proportional to the feedback error in the previous trial:

A46 $$ \begin{aligned} & \underline {v}_{ n + 1} = v_{ cn} + \kappa_{e} \cdot e_{n} \cdot \tilde{v}_{n} \; \\ & \quad {\text{with}}\;{ } v_{ cn} \sim N\left( {\underline {0} , \sigma^{2} } \right) \; {\text{and}}\;{ }\tilde{v}_{n} \sim N\left( {\underline {0} , 1} \right), \\ \end{aligned} $$

where the feedback error ${e}_{n}$ was defined according to Eq. (A10), Eq. (A13), and (A16f) by

A47 $$ e_{n} = \left\{ {\begin{array}{*{20}l} {u_{n} - \underline {c}_{ n}^{T} \underline {x}_{n} + \gamma_{n} v_{n} } \hfill & {{\text{for }}\;{\text{closed}}\;{\text{ loop}}} \hfill \\ {\delta_{n} + \gamma_{n} v_{n} } \hfill & {{\text{for}}\;{\text{ error}}\;{\text{ clamp}}} \hfill \\ \end{array} } \right. . $$

These definitions lead to the following difference equation for the temporal development of the execution noise:

A48 $$ \sigma_{n + 1}^{2} = \left\{ {\begin{array}{*{20}l} {\sigma^{2} + \kappa_{e}^{2} \left( {\left( {u_{n} - \underline {c}_{ n}^{T} \underline{{\overline{x}}}_{n} } \right)^{2} + \underline {c}_{n}^{T} {{\varvec{\Sigma}}}_{n} \underline {c}_{ n} + \gamma_{n}^{2} \sigma_{n}^{2} } \right)} \hfill & {\text{for closed loop}} \hfill \\ {\sigma^{2} + \kappa_{e}^{2} \left( {\delta_{n}^{2} + \gamma_{n}^{2} \sigma_{n}^{2} } \right)} \hfill & {\text{for error clamp}} \hfill \\ \end{array} } \right. . $$

Starting with ${\sigma }_{0}^{2}={\sigma }^{2}$, we applied Eq. (A48) to compute the time-dependent execution noise along with the iteration Eq. (A27a/b) for every *n* and used the result to replace the constant ${\sigma }^{2}$ in the generalized Kalman algorithm (Eq. 41a, A42c/f, and A44b) by the time-dependent ${\sigma }_{n}^{2}$.

The second type of model extension (M4) introduced a signal-dependent planning noise (${\underline{w}}_{n}$, Eq. A48) defined as the sum of a noise with constant covariance matrix ***W*** and a product of the respective component of state vector ${\underline{x}}_{n}$ with white Gaussian noise (${\stackrel{\sim }{\underline{w}}}_{n}$):

A49 $$ \begin{aligned} \underline {w}_{n} & = \underline {w}_{ cn} + diag\left( {\underline {\kappa } } \right) \cdot diag\left( {\underline {x}_{n} } \right) \cdot \widetilde{{\underline {w} }}_{n} \\ & \quad \quad {\text{with }}\;{ } \underline {w}_{ cn} \sim N\left( {\underline {0} , {\varvec{W}}} \right) {\text{and }}\widetilde{{\underline {w} }}_{n} \sim N\left( {\underline {0} , \left[ {\begin{array}{*{20}c} 1 & 0 \\ 0 & 1 \\ \end{array} } \right]} \right), \\ \end{aligned} $$

The random vector ${\stackrel{\sim }{\underline{w}}}_{n}$ was assumed to be independent of both the state vector ${\underline{x}}_{n}$ and the constant noise component ${\underline{w}}_{ cn}$. The vector $\underline{\kappa }=\left[{\kappa }_{s}; {\kappa }_{f}\right]$ denotes the two coefficients of variation of the slow and fast memory states. The time-dependent covariance matrix (${{\varvec{W}}}_{n}$) of this signal-dependent noise is

A50 $$ \begin{aligned} {\varvec{W}}_{n} & = {\varvec{W}} + diag\left( {\underline {\kappa } } \right)^{2} \cdot E\left\{ {diag\left( {\underline {x}_{n} } \right)^{2} } \right\} \\ & \quad = {\varvec{W}} + diag\left( {\underline {\kappa } } \right)^{2} \cdot \left( {diag\left( {diag\left( {{{\varvec{\Sigma}}}_{n} } \right)} \right) + diag\left( {\underline{{\overline{x}}}_{n} } \right)^{2} } \right). \\ \end{aligned} $$

The function *diag* is here defined as the MATLAB function *diag*, i.e., it returns a quadratic diagonal matrix with the elements of its argument in the diagonal if the argument is a vector and returns a vector with the diagonal elements of its argument if the argument is a quadratic matrix. Equation (A50) shows that ${{\varvec{W}}}_{n}$ depends only on $\underline{\kappa }$, ${\overline{\underline{x}}}_{n}$, and ${{\varvec{\Sigma}}}_{n}$. Moreover, the signal-dependent noise ${\underline{w}}_{n}$ is statistically independent of the state vector ${\underline{x}}_{n}$ (because of the assumed independence between ${\stackrel{\sim }{\underline{w}}}_{n}$ and ${\underline{x}}_{n}$). Consequently, in the recursion for computing the covariance matrix ***Y*** (Eq. A27b), the constant ***W*** can be substituted by the time-dependent ${{\varvec{W}}}_{n}$ as defined in Eq. (A50). It is important to note that the expectation $E\left\{diag{\left({\underline{x}}_{n}\right)}^{2}\right\}$ is taken in Eq. (A50) across all outputs $\underline{y}$ possibly observed for a given model input ${\left\{u\right\}}_{0}^{N-1}$ and fixed model parameters (${\varvec{A}},\underline{ b}, {\sigma }^{2}, {\varvec{W}},\boldsymbol{ }{\underline{\overline{x}}}_{0}, q, \underline{\kappa }$). Thus, the unconditioned mean ${\underline{\overline{x}}}_{n}$ and the unconditioned covariance matrix ${{\varvec{\Sigma}}}_{n}$ in Eq. (A50) are identical with those used in the recursion Eq. (A27a/b), but not with the state mean ${\underline{\overline{x}}}_{n}$ and the covariance matrix ${{\varvec{\Sigma}}}_{n}$ in the algorithm for the generalized Kalman observer (Eqs. A41–A45) because the later ones are conditioned on the observations ${\left\{y\right\}}_{0}^{n-1}$. To account for the time variant covariance ${{\varvec{W}}}_{n}$ of the state noise in the generalized Kalman observer, ${{\varvec{W}}}_{n}$ was computed in parallel with and before each step of the iteration Eq. (A27a/b). The resulting time variant ${{\varvec{W}}}_{n}$ was then used to replace the constant ${\varvec{W}}$ in the generalized Kalman algorithm (Eq. A44).

### A6: Model fitting

For each subject, the model parameters $\underline{\vartheta }=\left[{\varvec{A}}, \underline{b}, {\sigma }^{2}, {\varvec{W}}, {{\varvec{\Sigma}}}_{0}, {\underline{\overline{x}}}_{0}, q, {\kappa }_{s}, {\kappa }_{f}, {\kappa }_{e}\right]$ were fitted to the observed relative pointing directions ${\left\{y\right\}}_{0}^{N-1}$ by minimizing the negative log-likelihood $-log\left(L\left(\left.{\left\{y\right\}}_{0}^{N-1}\right|, \underline{{\vartheta }}\right)\right)$ under the following constraints:

1. The system (Eq. A16a–f) must be overdamped and asymptotically stable for both error-clamp trials and closed-loop trials, i.e., both eigenvalues of $${\varvec{A}}=\left[\begin{array}{cc}{a}_{s},& 0\\ 0,& {a}_{f}\end{array}\right]$$ as well as those of ${\varvec{A}}-\underline{b} {\underline{c}}^{T}$ must be real and in the interval between 0 and 1.
2. Both error sensitivities must be positive.
3. The initial value of the expected fast state was constrained to zero: $${\overline{\underline{x}}}_{0}=\left[\begin{array}{c}{\overline{x}}_{s0}\\ 0\end{array}\right]$$. This constraint was motivated by the observation that leaving both initial mean states as free parameters led to large ambiguity because large initial values of the fast state could be compensated by large initial values of the slow state without strong consequences on the likelihood. This ambiguity is the reason why both initial state values are difficult to estimate from the observation of the relative pointing directions only. This was already observed by Albert and Shadmehr (2017) who addressed this problem by using an expectation–maximization algorithm. Our approach to constrain the fast initial state to zero may provide a pragmatic and simple solution for this problem.
4. *q* must be in the interval between 0 and 1.
5. ${\sigma }^{2}$ must be positive.
6. ${\varvec{W}}$ is diagonal and positive-definite. Thus, we assumed that ${w}_{sf}^{2}$=0. The planning variances of the slow process (${w}_{ss}^{2}$>0) and of the fast process (${w}_{ff}^{2}$>0) were fitted.
7. In case of constant execution-noise variance ${\sigma }^{2}$ and constant planning-noise variances ${w}_{ss}^{2}$ and ${w}_{ff}^{2}$, the stability of the dynamics of the state mean (enforced by constraint 1) is sufficient to asure the stability of the linear time-discrete system Eq. (A27b) determining the temporal evolution of the planning variance ${{\varvec{\Sigma}}}_{n}$. In contrast, with signal-dependent planning noise or error-dependent execution noise, the coupling of the temporal evolution of ${{\varvec{\Sigma}}}_{n}$ (Eq. A27b) with that of ${{\varvec{W}}}_{n}$ (Eq. 50) or with that of ${\sigma }_{n}^{2}$ (Eq. A48) can become instable even when the constraint 1) is fulfilled. To avoid such an instability, further constraints were applied that were derived from the dynamics of the involved variances (${{\varvec{\Sigma}}}_{n}$, ${{\varvec{W}}}_{n}$) or (${{\varvec{\Sigma}}}_{n}$, ${\sigma }_{n}^{2}$). Equations (A27b/A50) or Eqs. (A27b/A48) show that they are linear with respect to the elements of ${{\varvec{\Sigma}}}_{n}$ and ${{\varvec{W}}}_{n}$. Therefore, they can be transformed into the standard linear form of a time-discrete dynamic state update. For the signal-dependent planning noise, we extract the elements of ${{\varvec{\Sigma}}}_{n}$ and ${{\varvec{W}}}_{n}$ (see Eqs. A8 and A20) in a single vector and obtain
  A51 $$ \left[ {\begin{array}{*{20}c} {\begin{array}{*{20}c} {w_{ss n + 1}^{2} } \\ {w_{ff n + 1}^{2} } \\ \end{array} } \\ {\begin{array}{*{20}c} {\sigma_{s n + 1}^{2} } \\ {\begin{array}{*{20}c} {\sigma_{f n + 1}^{2} } \\ {\sigma_{sf n + 1}^{2} } \\ \end{array} } \\ \end{array} } \\ \end{array} } \right] = {\varvec{M}}_{P} \left( {\underline {\kappa } ,{\varvec{A}}_{n} } \right)\left[ {\begin{array}{*{20}c} {\begin{array}{*{20}c} {w_{ss n}^{2} } \\ {w_{ff n}^{2} } \\ \end{array} } \\ {\begin{array}{*{20}c} {\sigma_{s n}^{2} } \\ {\begin{array}{*{20}c} {\sigma_{f n}^{2} } \\ {\sigma_{sf n}^{2} } \\ \end{array} } \\ \end{array} } \\ \end{array} } \right] + \underline {m}_{P} \left( {\underline {\kappa } , \underline{{\overline{x}}}_{n} , {\varvec{W}},\gamma_{n} , \sigma^{2} , \underline {b} } \right), $$
  where ${{\varvec{M}}}_{P}$ depends only on the coefficients of variation of the planning noise ($\underline{\kappa }$) and on ${{\varvec{A}}}_{n}$. The stability of this system was ensured by the constraint that the magnitude of the eigenvalues of the matrix ${{\varvec{M}}}_{P}$ must be smaller than one for $${{\varvec{A}}}_{{\varvec{n}}}=\left[\begin{array}{cc}{a}_{s},& 0\\ 0,& {a}_{f}\end{array}\right]$$ as well as for $${\varvec{A}}_{{\varvec{n}}} = \left[ {\begin{array}{*{20}c} {a_{s} ,} & 0 \\ {0,} & {a_{f} } \\ \end{array} } \right] - \underline {b} \underline {c}^{T}$$.
8. For error-dependent execution noise, the combined variance dynamics (Eqs. A27b/A48) takes the form
  A52 $$ \left[ {\begin{array}{*{20}c} {\sigma_{n + 1}^{2} } \\ {\sigma_{s n + 1}^{2} } \\ {\begin{array}{*{20}c} {\sigma_{f n + 1}^{2} } \\ {\sigma_{sf n + 1}^{2} } \\ \end{array} } \\ \end{array} } \right] = {\varvec{M}}_{{\varvec{M}}} \left( {\kappa_{e} ,\gamma_{n} ,{\varvec{A}}_{n} ,\underline {b} } \right)\left[ {\begin{array}{*{20}c} {\sigma_{n}^{2} } \\ {\sigma_{s n}^{2} } \\ {\begin{array}{*{20}c} {\sigma_{f n}^{2} } \\ {\sigma_{sf n}^{2} } \\ \end{array} } \\ \end{array} } \right] + \underline {m}_{M} \left( {\kappa_{e} , \underline{{\overline{x}}}_{n} , u_{n} ,\delta_{n} , {\varvec{W}}, \sigma^{2} } \right). $$
  To ensure the variance stability in the case of error proportional execution noise, we constrained the magnitude of the eigenvalues of ${{\varvec{M}}}_{M}$ to stay below one for any of the occurring values of ${\gamma }_{n}$ and ${{\varvec{A}}}_{n}$.
9. The initial value ${{\varvec{\Sigma}}}_{0}$ of the state covariance matrix was assumed to be identical with its asymptotic value (${{\varvec{\Sigma}}}_{\infty }$) for the initial trial type, i.e., closed-loop trials in our experiment. For models without signal-dependent noise (${\kappa }_{s}={\kappa }_{f}={\kappa }_{e}=0$), we computed ${{\varvec{\Sigma}}}_{0}={{\varvec{\Sigma}}}_{\infty }$ according to Eq. (A29) for any given ${\varvec{A}}$, $\underline{b}$, ${\sigma }^{2}$, ${\varvec{W}}$, and $q$. For models with signal-dependent noise, ${{\varvec{\Sigma}}}_{0}={{\varvec{\Sigma}}}_{\infty }$ was computed from the stationary solution of Eq. (A51) or from that of Eq. (A52). Consequently, the elements ${{\varvec{\Sigma}}}_{0}$ were not treated as additional model parameters to be fitted.

All models investigated in this study were fitted under the above constraints and always fitted the 7 parameters${x}_{s0}$,${a}_{s}$, ${a}_{f},{b}_{s}$,${b}_{f}$,$q$, and${\sigma }^{2}$. They differed only with respect to additional constraints on the variance parameters${w}_{ss}^{2}$,${w}_{ff}^{2}$,${\kappa }_{s}$,${\kappa }_{f}$, and ${\kappa }_{e}$.

The computation of the negative log-likelihood as a function of the model parameters $\underline{\vartheta }$, based on the generalized Kalman observer, was implemented by a custom code written in MATLAB and numerically minimized by using the MATLAB function *fmincon*. The code is available at https://github.com/T-Eggert/ClosedLoopSystemIdentification

## Footnotes

1 Minimizing the mean-squared residual and minimizing the likelihood are identical for a variance model predicting white and Gaussian residuals.

## Code availability

The code is available at https://github.com/T-Eggert/ClosedLoopSystemIdentification.
